# Supplementary material for: Co‐targeting BET and MEK as salvage therapy for MAPK and checkpoint inhibitor‐resistant melanoma
Source: EMBO Mol Med. 2018 Apr 11;10(5):e8446. doi: 10.15252/emmm.201708446 (PMC5938620; doi:10.15252/emmm.201708446)
Supplement: Supplementary file 1 — Appendix [file EMMM-10-e8446-s001.pdf]

APPENDIX

CO-TARGETING BET AND MEK AS SALVAGE THERAPY FOR MAPK AND CHECKPOINT INHIBITOR RESISTANT MELANOMA

Ileabett M. Echevarría-Vargas[1], Patricia I. Reyes-Uribe[1], Adam N. Guterres[1], Xiangfan Yin[1], Andrew V. Kossenkov[1], Qin Liu[1], Gao Zhang[1], Clemens Krepler[1], Chaoran Cheng[3], Zhi Wei[3], Rajasekharan Somasundaram[1], Giorgos Karakousis[4,5], Wei Xu[4], Jennifer J.D. Morrissette[6,7], Yiling Lu[8], Gordon B. Mills[8], Ryan J. Sullivan[9], Miao Benchun[9], Dennie T. Frederick[9], Genevieve Boland[10], Keith T. Flaherty[9], Ashani T. Weeraratna[10,11], Meenhard Herlyn[1,10], Ravi Amaravadi[4,12], Lynn M. Schuchter [4,12], Christin E. Burd[13], Andrew E. Aplin[14], Xiaowei Xu[4,7], and Jessie Villanueva[1,10*].

TABLE OF CONTENTS

Appendix Materials and Methods

Appendix Figure Legends

Appendix Figures 1-9

Appendix Tables 1-8

APPENDIX MATERIALS AND METHODS

Western blot analysis

Cells were collected and washed twice with ice-cold Phosphate Buffer Saline (PBS), harvested and stored at -80°C until processed. Total cell lysates were prepared as previously described (Villanueva *et al*, 2010). Protein concentration was determined using Bio-Rad Protein Assay (Bio-Rad, Hercules, CA). Protein lysates (30-50 µg) were separated by SDS-PAGE, blotted onto nitrocellulose membranes, and probed with primary antibodies (Appendix Table S8). Primary antibodies used were: anti-MYC (5605), anti-caspase-7 cleaved (9491T), anti-caspase-3 cleaved (9661), anti-pRB S807/811 (9308), anti-PLK1 (4535), anti-AuroraK B (3094), anti-phospho ERK1/2 (1:5000; 4370), and anti-cleaved PARP (5625) (Cell Signaling, Beverly, MA). All antibodies from Cell Signaling were used at 1:1000 dilutions, except when otherwise noted. Other antibodies used were: anti-BRD2 (1:1000; A302-582A), anti-BRD3 (1:1000; A302-368A), and anti-BRD4 (1:1000; A301-985A) (Bethyl Laboratories, Montgomery, TX); anti-CyclinD1 (1:1000; 04-1151) (Millipore, Billerica Massachusetts); anti-BIRC5 (1:1000; NB500-201) (Novus Biologicals LCC, Littleton, CO); anti-BIM (1:1000; ab32158), anti-TCF19 (1:500; ab57681) (Abcam Inc, Boston, MA); anti-TCF19 (1:500; sc-101169) (Santa Cruz, Dallas, TX); anti-TCF19 (1:1000; TA333897) (OriGene Technologies, Inc, Rockville, MD); and anti-βactin (1:5000; A5441) (Sigma-Aldrich, St. Louis, MO). Secondary antibodies used were IRDye 680RD Goat anti-mouse (926-68070) and IRDye 800CW Goat anti-rabbit (926-32211), both used at 1:10000 dilutions (LI-COR biosciences, Lincoln, NE). Blots were scanned and analyzed using the ODYSSEY Infrared Imaging System application software version 3.0 (LI-COR biosciences, Lincoln, NE).

BRD4 and TCF19 short hairpin RNAs, and TCF19-ORF

Lentiviral-encoded shRNAs from the TRC shRNA library (Thermo Fisher Scientific, Waltham, MA) were used to silence BRD4 (TRCN0000021424 (sh-#4) and TRCN0000021427 (sh-#7) and TCF19 (TRCN0000235708 (sh-#UTR) and TRCN0000015411 (sh-#11). TCF19 was expressed via a lentivirally packaged pLX304 ORF expression vector (TOLH-1516351) (transOMIC technologies, Hunstville, AL). Cells were transduced as previously described (Villanueva *et al*, 2010); transduced cells were selected with 2µg/ml puromycin for 48h and/or 8µg/ml blasticidin.

Crystal Violet assays

1 x 10^4 cells were seeded onto 6-well cell culture plates and treated with 0.5µM JQ-1, 0.1µM PD901, or the combination of the two drugs (JQ-1 + PD901) for seven or fourteen days. Cells were fixed with 3.7% formaldehyde for 10 min, and stained with 0.5% crystal violet dye in 20% methanol for 2 hours at room temperature. Excess crystal violet dye was removed by three washes with PBS, and the culture plates were dried overnight. The crystal violet stain was eluted with 0.1M NaCitrate, pH 4.2 in 50% ethanol. Optical density (OD; 595 nm) was measured using a microplate reader (BioTek Elx800).

Colony formation assay

M93-047 (1.25 x 10^2) cells were seeded onto 100 mm culture plates and treated with 0.5µM JQ-1, 0.1µM PD901, or combination (JQ-1 + PD901) for fourteen days. Cells were fixed with 3.7% formaldehyde for 10 min and stained with 0.5% crystal violet dye in 20% methanol for 2 hours at room temperature. Excess crystal violet dye was removed by three washes with PBS, and the culture plates were dried overnight. Cells were imaged using a digital camera and number of colonies was determined using ImageJ program.

RNA-sequencing

Total RNA was isolated using the *PureLink RNA Mini Kit* (Life Technology, Grand Island, NY). RNA underwent quality control using the Agilent Bioanalyzer (Agilent, Santa Clara, CA). Poly-A library was prepared using Lexogen QuantSeq 3'mRNA-Seq library prep kit at the Wistar Institute Genomic Core. The prepared library was sequenced using Illumina Next-Generation sequencing (NextSeq 500) using the Mid Output V2 (150 cycles) kit (cat no. FC-404-2001) with sequences produced in 1x 75 base pair run. Raw reads were trimmed from poly-A tails and aligned using bowtie 2 algorithm (Langmead & Salzberg, 2012) against hg19 genome, and Ensembl transcriptome information was used along with RSEM software (Frezza *et al*, 2011) in order to calculate raw read counts for each gene. Differential expression analysis between pairs of groups was done using DESeq2 (Love *et al*, 2014). Genes that passed the FDR threshold <5% were considered significant and genes that were found significantly affected by both combo *vs.* DMSO and combo *vs.* single agent treatment were used in the final gene set. Analysis of gene set for enrichment of biological functions was performed using QIAGEN's Ingenuity Pathway Analysis software (IPA, QIAGEN Redwood City; www.qiagen.com/ingenuity). Only significant results (FDR<5% for pathways, $p<10^{-4}$ for functions) with Z-score for predicted activation state calculated by IPA of at least 2 were considered. RNA-seq data are available in Gene Expression Omnibus (accession number GSE95153; https://www.ncbi.nlm.nih.gov/geo/query/acc.cgi?token=wxydcyeezbknbcx&acc=GSE95153).

RNA was extracted from patient tumor samples (obtained from MGH Cancer Center) with Qiagen RNeasy Mini kit. RNA libraries were prepared from 250 ng RNA using standard Illumina protocols. RNA sequencing was performed at the Broad Institute (Illumina HiSeq2000) and the Wistar Institute Genomic Core Facility (Illumina NextSeq 500). Raw

RNA-Seq data (BAM files) read counts were summarized by featureCounts with parameters that only paired-ended, not chimeric and well mapped (mapping quality >= 20) reads were counted. Normalization was applied to eliminate bias from sequencing depths and gene lengths by edgeR, RPKMs (Reads Per Kilobase of transcript per Million mapped reads) were used for analysis.

Real-time qPCR

Total RNA was isolated using PureLink RNA Mini kit and cDNA was synthesized using Maxima First Strand cDNA Synthesis kit (ThermoFisher Scientific). Total RNA (1 µg), 2 µl maxima enzyme, and 4µl 5X reaction mix were mixed in a final volume of 20 µL. Samples were mixed, centrifuged and incubated at 25°C for 10 min, 50°C for 15 min and 85°C for 5 min. SYBR-I-real time PCR were performed using Fast SYBR-Green Master Mix (Applied Biosystems) in a StepOne plus real-time PCR thermal cycle system (Applied Biosystems). The PCR reaction mix contained 10 µl Fast SYBRGreen Master Mix, 0.5 µl forward primer, 0.5 µL reverse primer, 2µL cDNA, and 7 µL of water (Appendix Table S7). Cycling conditions: one cycle of 10 min at 95°C, and 40 cycles of 15 sec at 95°C, 1 min at 60°C. Melt curve analysis was performed at the end of each PCR reaction. Relative gene expression was calculated with the ΔΔCt-method (Schmittgen & Livak, 2008).

Reverse phase protein array

Cells ($1x10^6$) were treated with DMSO, JQ-1, PD901, or combo. Forty-eight hours later, cells were collected, and protein extracts were prepared using lysis buffer (1X TNE pH 8.0, 1% NP40, X mM NaV, 1X protease inhibitor cocktail, 5mM Tris-HCl pH 7.4, 15 mM NaCl) and 1% SDS. The RPPA was performed at the MD Anderson Cancer Center RPPA Core Facility. Briefly, samples were arrayed on nitrocellulose-coated slides, probed with 304 antibodies detected by 3,3-diaminobenzidine (DAB) colorimetric reaction, scanned,

analyzed, and quantified using MicroVigene software (VigeneTech Inc.). The analysis was performed using the R package SuperCurve, developed by the Department of Bioinformatics and Computational Biology at University of Texas MD Anderson Cancer Center (Tibes *et al*, 2006).

IPRES ssGSEA analysis

Single-sample gene set enrichment (ssGSEA) was performed using the GSVA package in R to derive the enrichment scores of the IPRES gene signatures (Durinck *et al*, 2009). GSVA score for each gene signature in each sample was derived using the biomatRt package in R to match the ensemble gene id symbol (Durinck *et al*, 2005; Hänzelmann *et al*, 2013) and the record with maximum variance was kept for same gene symbol if multiple records exist. Normalized log2 RPKM values were passed on as input for GSVA.

APPENDIX FIGURES LEGENDS

Appendix Figure S1. BRD4 expression does not correlate with clinical outcomes in BRAF^{mut}, NF1^{mut} or triple WT melanoma patients.

BRD-4, -3, and -2 mRNA expression for different genetic [BRAF-mutant, NF1-mutant and WT/WT/WT] melanoma cohorts were downloaded from TCGA skin cutaneous melanoma dataset and stratified into two groups (BRD-low or BRD-high) according to the median tissue mRNA expression levels.

A-B. BRAF-mutant (n = 188)

C-D. NF1-mutant (n = 47)

E-F. WT/WT/WT (n = 170)

Correlation between BET proteins (BRD4, 3, and 2) and overall survival **(A, C, and E)** or disease-free survival **(B, D, and F)** was determined using the Kaplan-Meier method. P-values were calculated by long-rank test comparing the two Kaplan Meier curves.

Appendix Figure S2. Lentiviral mediated depletion of BRD4.

BRD4 was silenced using two different hairpins (#4 and #7) in NRAS mutant melanoma cells (FS13, M93-047, and WM3000).

A. Total cell lysates were analyzed by immunoblotting; β-actin was used as loading control. Representative western blots are shown. Protein levels (shown below each band) were quantified using the Li-COR Odyssey system, normalized to its corresponding β-actin, and calculated relative to empty vector-transduced cells (EV).

B. Average relative BRD4 and BRD2 protein levels assessed by immunoblotting from three independent experiments +/- SEM is shown.

Appendix Figure S3. Co-targeting BET and MEK induces apoptosis in NRAS mutant melanoma cells.

NRAS mutant melanoma cells M93-047, WM3000 and WM852 were treated with DMSO (vehicle control), the BETi OTX-015 (0.5 μM), the MEKi trametinib (0.1 μM) or the combination of both drugs at the same doses. After 7 days of treatment, cells were stained with Annexin V/PI and cell death was assessed and quantified by FACS. Percent of cells Annexin V+/PI+ is shown. Data represent the mean of three independent experiments +/- SEM. Statistically significant differences were determined by Student's t-test.

Appendix Figure S4. Combining JQ-1 with CDK4/6 or PI3K inhibitors impairs cell viability.

NRAS mutant melanoma cells were treated with JQ1 in combination with PD0332991 or BKM120.

A. Cell viability was determined by Alamar Blue assay after five days of treatment. Interaction index and 95% confidence interval (CI) for each cell line is shown. The upper limit of its 95% CI < 1 was considered significant synergy.

B-C. Cells were seeded in 12-well plates and treated with a single dose of 0.5µM JQ-1, 0.1µM PD991 **(B)**, 0.1µM BKM120 **(C)**, combination or DMSO. Cells were fixed after 7 or 14 days and stained with crystal violet and relative number of cells quantified. Data represent the average of three independent experiments -/+ SEM. P values were calculated by Student's t-test.

Appendix Figure S5. Combination of BETi JQ-1/OTX-015 and PD0325901 does not affect animal weight.

A-B. Mice were treated with vehicle, JQ-1 (**A**; 25 mg/kg ip.qd), OTX-015 (**B**; 25 mg/kg po.qd), PD901 (5 mg/kg po.qd), or combinations of BET and MEK inhibitors. Xenografts were treated for 21 days. Average weight +/- SEM of mice included in the studies *vs.* time is shown.

Appendix Figure S6. Validation of genes involved in cell cycle regulation, DNA replication, and apoptosis.

A-**B**. Cells were treated with a single dose of 0.5µM JQ-1, 0.1µM PD901, combo, or DMSO. After 48 hours, cells were collected, RNA was extracted and mRNA levels were quantified by qRT-PCR. Average values of three independent experiments are shown +/- SEM.

C. TCF19, E2F1 and E2F3 mRNA levels were determined by qRT-PCR in M93-047, WM3000, and WM852 cell lines. Ct values were normalized to endogenous β-actin. Data represent the average of three independent experiments +/- SEM.

A-C. P Values were calculated by Student t-Test and are shown in Appendix Table S3

D. NRAS mutant melanoma cells were transduced with BRD4 shRNA lentivirus. Three days post transduction; cells were treated with the MEKi trametinib (100 nM) (+) or DMSO (-) for additional 24h. Cells were collected, lysed and analyzed by immunoblotting with the indicated antibodies. Membranes were scanned and quantified using the LiCor Odyssey system. Numbers below each lane indicate relative band intensity normalized to the actin loading control.

E. Proteins identified by RPPA were validated by immunoblotting in NRAS mutant melanoma cells (M93-047, WM3000, and WM852) treated with vehicle, OTX-015, PD901, or combination for 48 hours. Proteins were quantified using the LiCor Oddysey system and normalized using actin as loading control. Protein levels relative to DMSO are shown below each band.

Appendix Figure S7. Depletion of TCF19 causes accumulation of cells in G2/M.

NRAS mutant melanoma cells M93-047 were transduced with vector control (EV) or two different TCF19 shRNAs (shUTR or sh11). Three days following transduction cells were collected, fixed and stained with propidium iodide (PI) and analyzed by FACS. Percent of cells in each phase of the cell cycle is shown.

Appendix Figure S8. BRD4 and TCF19 expression levels are associated in melanoma. RNA-seq data from TCGA database for Skin Cutaneous Melanoma set (n = 472) was downloaded and normalized using EdgeR method. NRAS and BRAF mutation status for

368 samples with whole exome sequencing data was downloaded from cBioPortal.org and

samples were stratified based on genetic mutations: NRASmut, BRAFmut, WT/WT. Correlation of normalized mRNA expression levels between BRD4 and TCF19 for NRASmut, BRAFmut, WT/WT and all samples together was performed using Spearman non-parametric test.

Appendix Figure S9. BETi/MEKi combo does not significantly affect immune cells *in vivo*.

A-D. BRAF-inhibitor resistant Yumm1.7-BR cells ($2x10^5$) were injected subcutaneously into 5-week-old C57Bl/6 mice. Mice were randomized into two treatment groups: vehicle control (n = 7) or combo (OTX-015 + PD901 x 12 days, n = 8). T cells were isolated from tumors (**A**) and spleens (**B**), and live cells gated by zombie yellow exclusion staining. $CD4^+$, $CD8^+$ and $CD152^+$/$Foxp3^+$ cells were assessed by flow cytometry. Myeloid cells and macrophages were isolated from tumors (**C**) and spleens (**D**) and live cells gated using DAPI exclusion. M-MDSCs, PMN-MDSCs and macrophages were assessed using $Ly6C^+$, $Ly6G^+$ and $F4/80^+$ respectively. Statistically significant differences were assessed by Student's t-test.

E. NRAS mutant WHN89 tumors were implanted subcutaneously into the flank of 5-week-old C57Bl/6 mice. Mice were randomized into five treatment groups: vehicle control (n = 8), anti-PD-1 (300 mg every five days, n = 8), OTX-015 (25 mg/kg po.qd, n = 8), PD901 (5 mg/kg po.qd, n = 8) or combination (n = 13). Tumor volume was measured by caliper every three days. P values were calculated by Student's t-Test

F. TCF19 protein levels were assessed by immunoblotting in tumors derived from LSL-$NRAS^{Q61R}$ mice treated for 13 days. Protein levels (shown below each band) were quantified using the Li-COR Odyssey system, normalized to its corresponding β-actin loading control.

G. Short-term cultures (13-456-5-3 and WM4231-2) derived from immunotherapy resistant patients were treated with 0.5μM OTX-015, 0.1 μM PD901, or combination (OTX-015/PD901) for 48 hours. Protein expression was assessed by immunobloting; β-actin and vinculin were used as loading control.

REFERENCES

Durinck S, Moreau Y, Kasprzyk A, Davis S, De Moor B, Brazma A & Huber W (2005) BioMart and Bioconductor: a powerful link between biological databases and microarray data analysis. *Bioinformatics* **21:** 3439–40

Durinck S, Spellman PT, Birney E & Huber W (2009) Mapping identifiers for the integration of genomic datasets with the R/Bioconductor package biomaRt. *Nat. Protoc.* **4:** 1184–91

Frezza C, Zheng L, Folger O, Rajagopalan KN, MacKenzie ED, Jerby L, Micaroni M, Chaneton B, Adam J, Hedley A, Kalna G, Tomlinson IPM, Pollard PJ, Watson DG, Deberardinis RJ, Shlomi T, Ruppin E & Gottlieb E (2011) Haem oxygenase is synthetically lethal with the tumour suppressor fumarate hydratase. *Nature* **477:** 225–8

Hänzelmann S, Castelo R & Guinney J (2013) GSVA: gene set variation analysis for microarray and RNA-seq data. *BMC Bioinformatics* **14:** 7

Langmead B & Salzberg SL (2012) Fast gapped-read alignment with Bowtie 2. *Nat. Methods* **9:** 357–9

Schmittgen TD & Livak KJ (2008) Analyzing real-time PCR data by the comparative C(T) method. *Nat. Protoc.* **3:** 1101–8

Tibes R, Qiu Y, Lu Y, Hennessy B, Andreeff M, Mills GB & Kornblau SM (2006) Reverse phase protein array: validation of a novel proteomic technology and utility for analysis of primary leukemia specimens and hematopoietic stem cells. *Mol. Cancer Ther.* **5:** 2512–21

Villanueva J, Vultur A, Lee JT, Somasundaram R, Fukunaga-Kalabis M, Cipolla AK, Wubbenhorst B, Xu X, Gimotty PA, Kee D, Santiago-Walker AE, Letrero R, D'Andrea K, Pushparajan A, Hayden JE, Brown KD, Laquerre S, McArthur GA, Sosman JA, Nathanson KL, et al (2010) Acquired resistance to BRAF inhibitors mediated by a RAF kinase switch in melanoma can be overcome by cotargeting MEK and IGF-1R/PI3K. Cancer Cell **18:** 683–95

S1

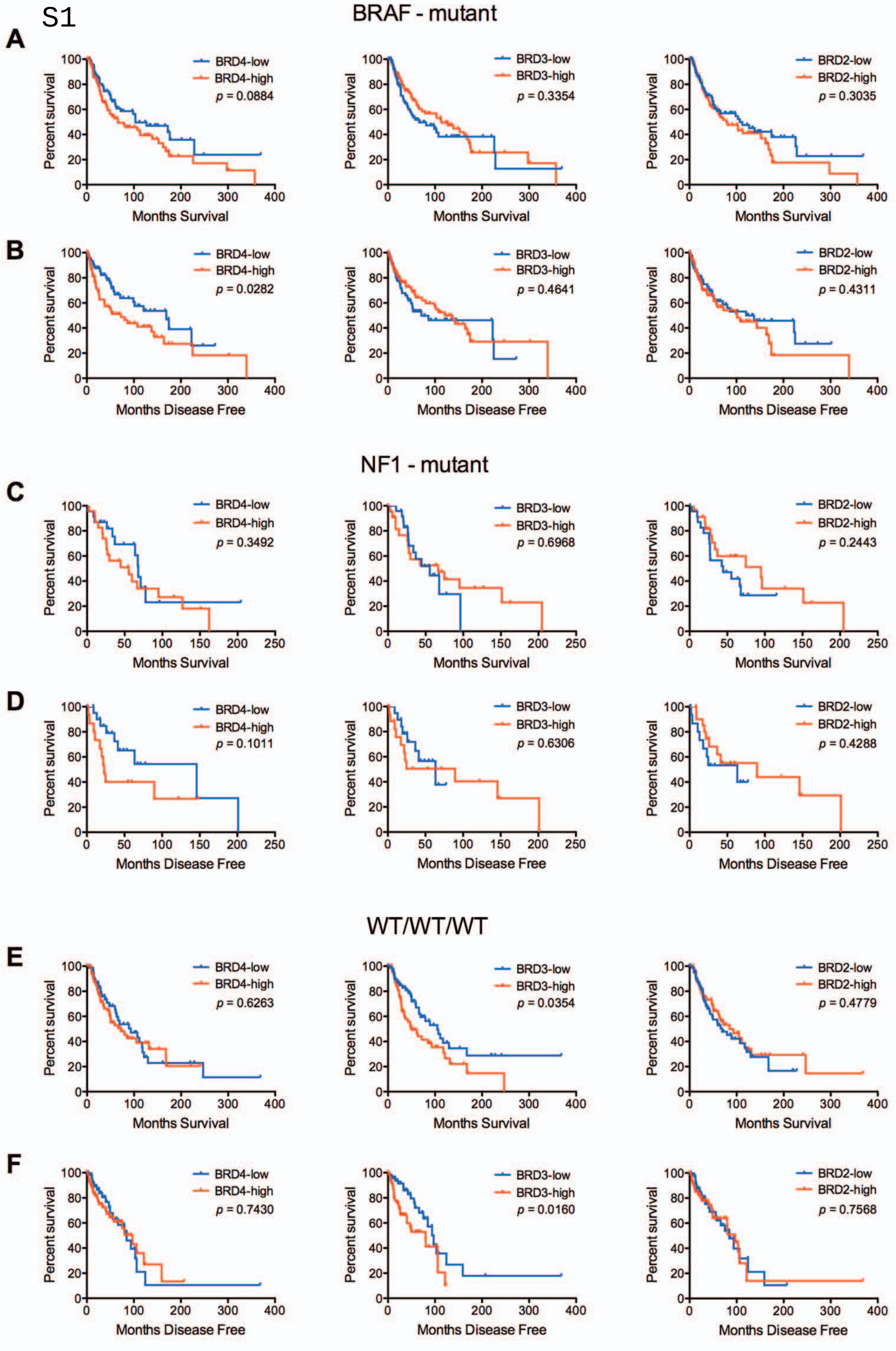
BRAF - mutant
A
BRD4-low
BRD4-high
p = 0.0884
BRD3-low
BRD3-high
p = 0.3354
BRD2-low
BRD2-high
p = 0.3035
Percent survival
Months Survival
B
BRD4-low
BRD4-high
p = 0.0282
BRD3-low
BRD3-high
p = 0.4641
BRD2-low
BRD2-high
p = 0.4311
Percent survival
Months Disease Free
NF1 - mutant
C
BRD4-low
BRD4-high
p = 0.3492
BRD3-low
BRD3-high
p = 0.6968
BRD2-low
BRD2-high
p = 0.2443
Percent survival
Months Survival
D
BRD4-low
BRD4-high
p = 0.1011
BRD3-low
BRD3-high
p = 0.6306
BRD2-low
BRD2-high
p = 0.4288
Percent survival
Months Disease Free
WT/WT/WT
E
BRD4-low
BRD4-high
p = 0.6263
BRD3-low
BRD3-high
p = 0.0354
BRD2-low
BRD2-high
p = 0.4779
Percent survival
Months Survival
F
BRD4-low
BRD4-high
p = 0.7430
BRD3-low
BRD3-high
p = 0.0160
BRD2-low
BRD2-high
p = 0.7568
Percent survival
Months Disease Free

S2

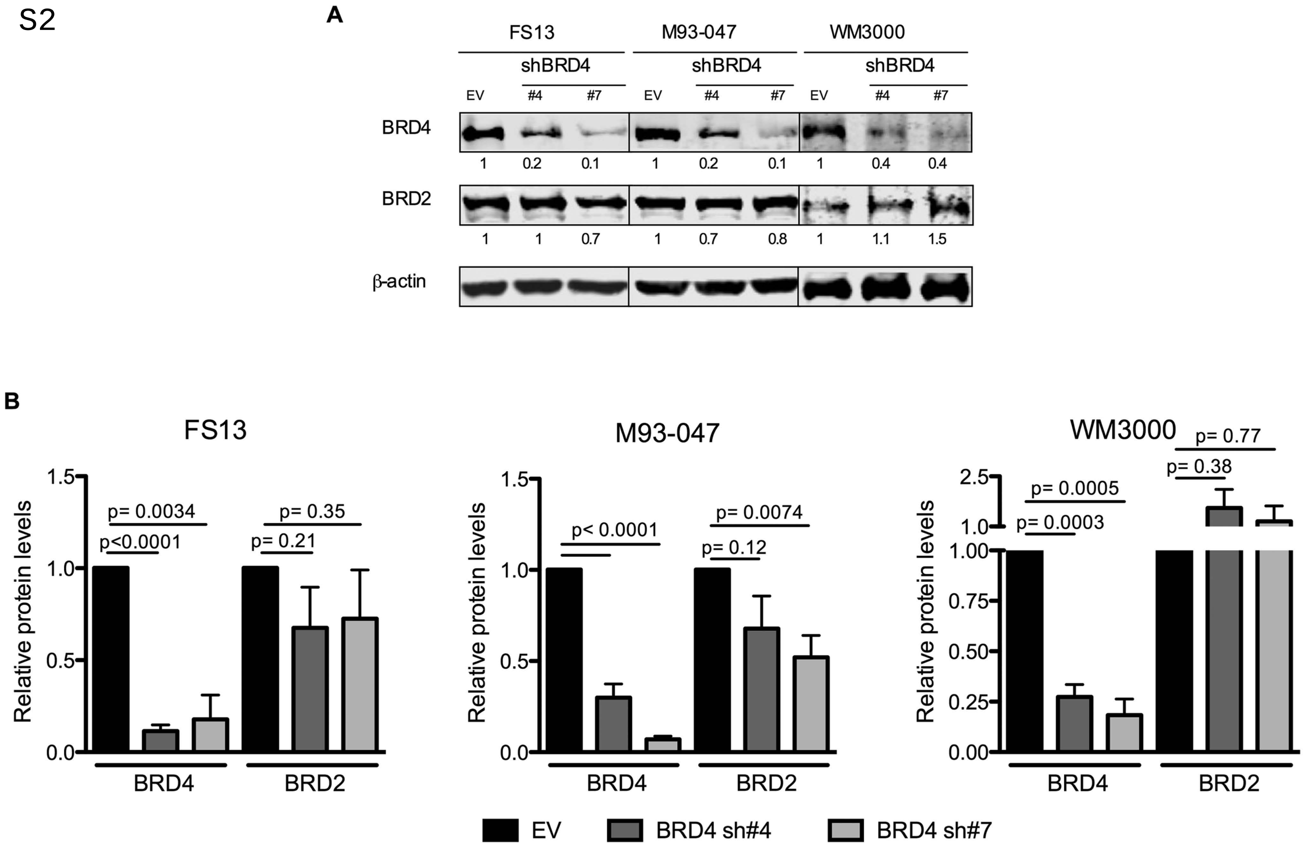
A
FS13
M93-047
WM3000
shBRD4
EV
#4
#7
BRD4
1 0.2 0.1 1 0.2 0.1 1 0.4 0.4
BRD2
1 1 0.7 1 0.7 0.8 1 1.1 1.5
β-actin
B
FS13
M93-047
WM3000
Relative protein levels
p<0.0001
p= 0.0034
p= 0.21
p= 0.35
p< 0.0001
p= 0.12
p= 0.0074
p= 0.0003
p= 0.0005
p= 0.38
p= 0.77
BRD4
BRD2
EV
BRD4 sh#4
BRD4 sh#7

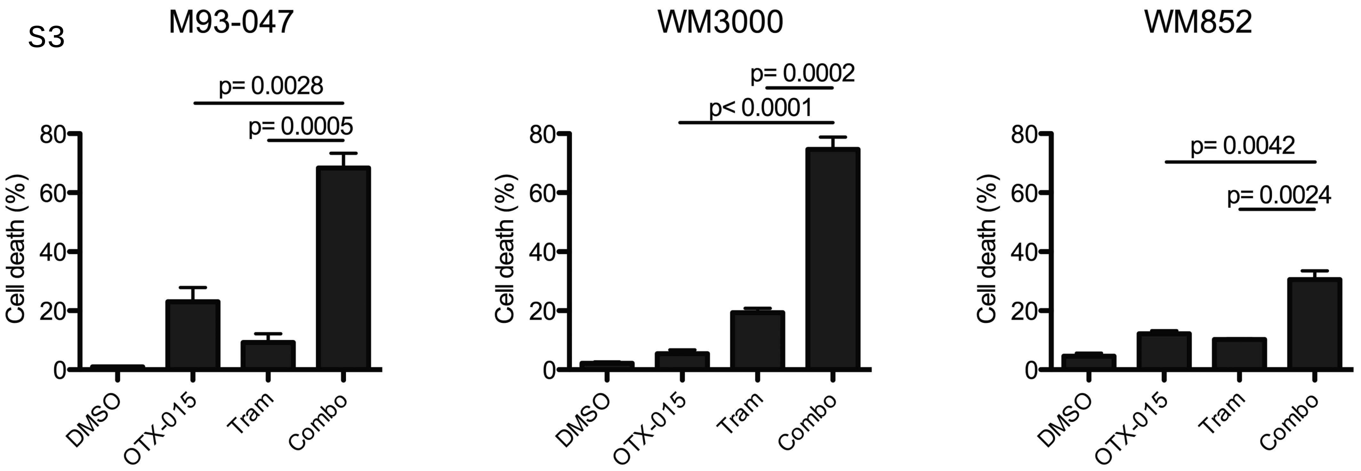
S3
M93-047
p= 0.0028
p= 0.0005
Cell death (%)
80
60
40
20
0
DMSO
OTX-015
Tram
Combo
WM3000
p= 0.0002
p< 0.0001
Cell death (%)
80
60
40
20
0
DMSO
OTX-015
Tram
Combo
WM852
p= 0.0042
p= 0.0024
Cell death (%)
80
60
40
20
0
DMSO
OTX-015
Tram
Combo

A S4

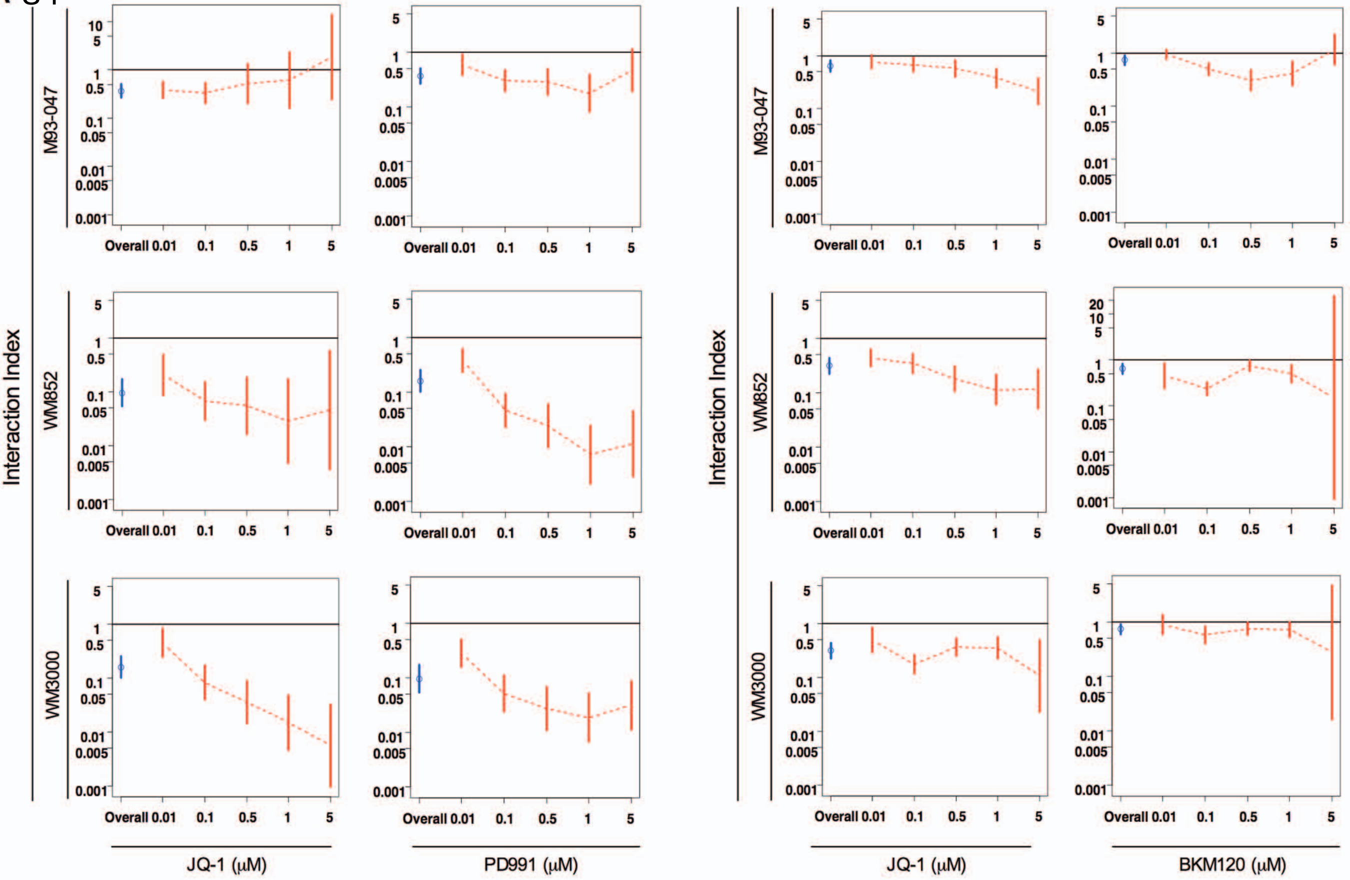
Interaction Index
M93-047
WM852
WM3000
Overall
0.01
0.1
0.5
1
5
JQ-1 (μM)
PD991 (μM)
BKM120 (μM)

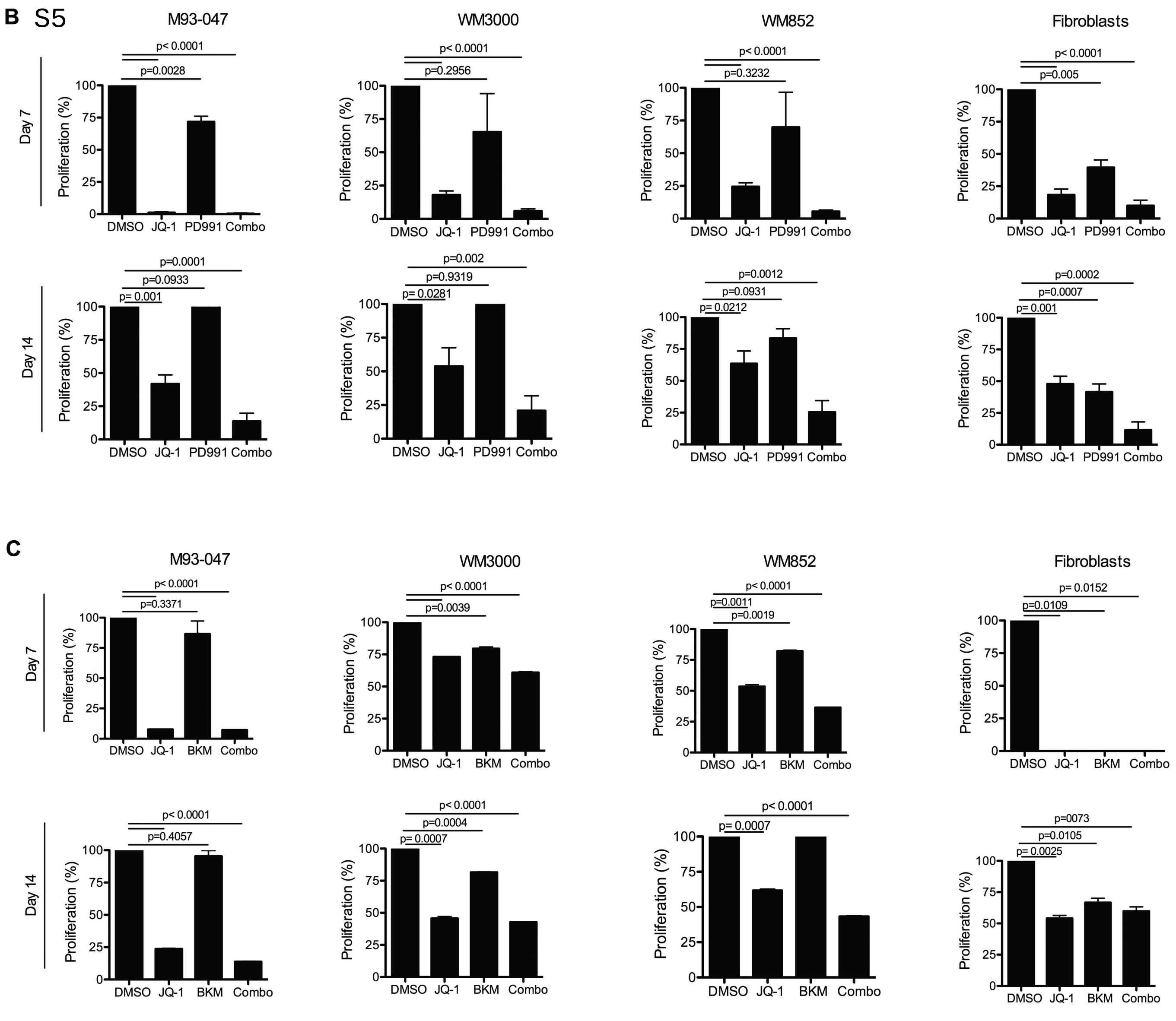

B S5
M93-047
WM3000
WM852
Fibroblasts
Day 7
Day 14
Proliferation (%)
DMSO JQ-1 PD991 Combo
p< 0.0001
p=0.0028
p=0.2956
p=0.3232
p=0.005
p=0.0001
p=0.0933
p= 0.001
p=0.002
p=0.9319
p= 0.0281
p=0.0012
p=0.0931
p= 0.0212
p=0.0002
p=0.0007
p= 0.001
C
DMSO JQ-1 BKM Combo
p=0.3371
p=0.0039
p=0.0011
p=0.0019
p= 0.0152
p=0.0109
p=0.4057
p=0.0004
p= 0.0007
p=0073
p=0.0105
p= 0.0025

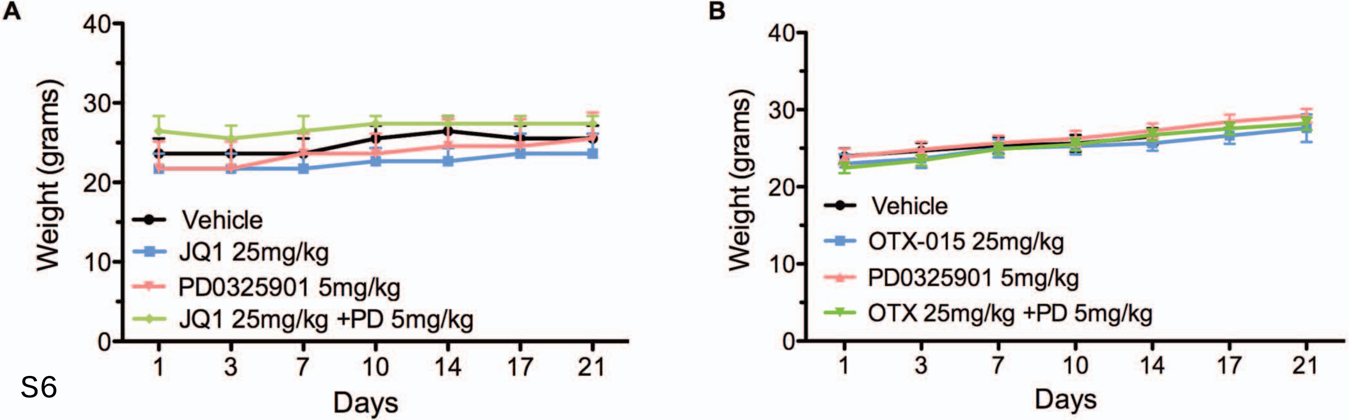
A
40
30
20
10
0
Weight (grams)
Vehicle
JQ1 25mg/kg
PD0325901 5mg/kg
JQ1 25mg/kg +PD 5mg/kg
1
3
7
10
14
17
21
Days
B
40
30
20
10
0
Weight (grams)
Vehicle
OTX-015 25mg/kg
PD0325901 5mg/kg
OTX 25mg/kg +PD 5mg/kg
1
3
7
10
14
17
21
Days

S6

S7

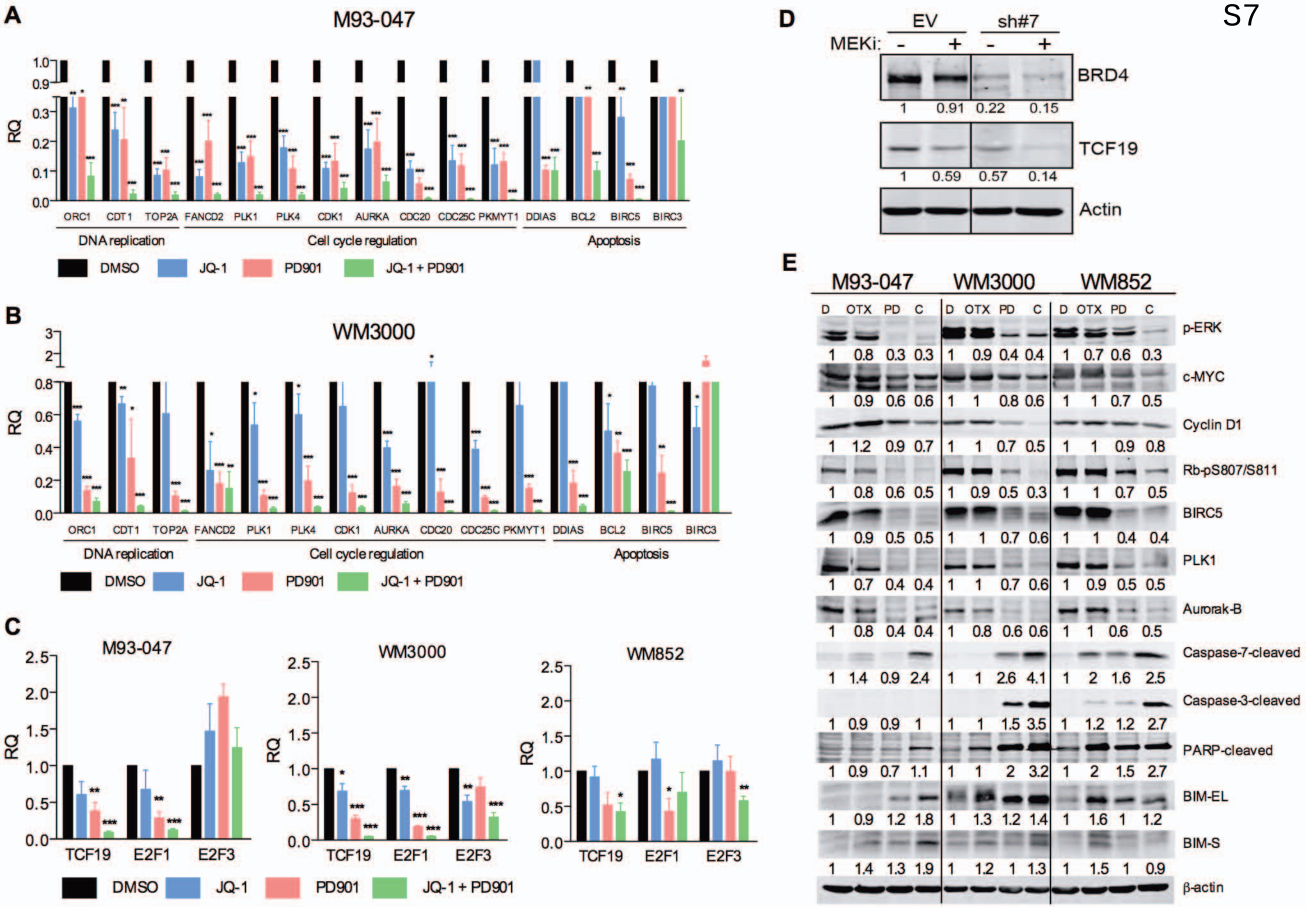
A
M93-047
RQ
1.0
0.9
0.3
0.2
0.1
0.0
ORC1 CDT1 TOP2A FANCD2 PLK1 PLK4 CDK1 AURKA CDC20 CDC25C PKMYT1 DDIAS BCL2 BIRC5 BIRC3
DNA replication
Cell cycle regulation
Apoptosis
DMSO JQ-1 PD901 JQ-1 + PD901
B
WM3000
RQ
3
2
0.8
0.6
0.4
0.2
0.0
ORC1 CDT1 TOP2A FANCD2 PLK1 PLK4 CDK1 AURKA CDC20 CDC25C PKMYT1 DDIAS BCL2 BIRC5 BIRC3
DNA replication
Cell cycle regulation
Apoptosis
DMSO JQ-1 PD901 JQ-1 + PD901
C
M93-047
WM3000
WM852
RQ
2.5
2.0
1.5
1.0
0.5
0.0
TCF19 E2F1 E2F3
DMSO JQ-1 PD901 JQ-1 + PD901
D
EV
sh#7
MEKi: - + - +
BRD4
1 0.91 0.22 0.15
TCF19
1 0.59 0.57 0.14
Actin
E
M93-047 WM3000 WM852
D OTX PD C D OTX PD C D OTX PD C
p-ERK
1 0.8 0.3 0.3 1 0.9 0.4 0.4 1 0.7 0.6 0.3
c-MYC
1 0.9 0.6 0.6 1 1 0.8 0.6 1 1 0.7 0.5
Cyclin D1
1 1.2 0.9 0.7 1 1 0.7 0.5 1 1 0.9 0.8
Rb-pS807/S811
1 0.8 0.6 0.5 1 0.9 0.5 0.3 1 1 0.7 0.5
BIRC5
1 0.9 0.5 0.5 1 1 0.7 0.6 1 1 0.4 0.4
PLK1
1 0.7 0.4 0.4 1 1 0.7 0.6 1 0.9 0.5 0.5
Aurorak-B
1 0.8 0.4 0.4 1 0.8 0.6 0.6 1 1 0.6 0.5
Caspase-7-cleaved
1 1.4 0.9 2.4 1 1 2.6 4.1 1 2 1.6 2.5
Caspase-3-cleaved
1 0.9 0.9 1 1 1 1.5 3.5 1 1.2 1.2 2.7
PARP-cleaved
1 0.9 0.7 1.1 1 1 2 3.2 1 2 1.5 2.7
BIM-EL
1 0.9 1.2 1.8 1 1.3 1.2 1.4 1 1.6 1 1.2
BIM-S
1 1.4 1.3 1.9 1 1.2 1 1.3 1 1.5 1 0.9
β-actin

S8

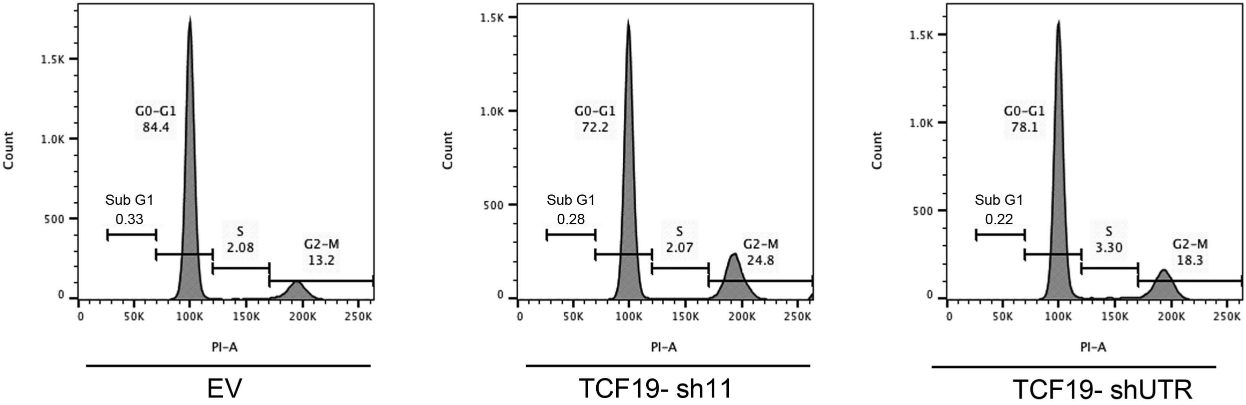

Count
1.5K
1.0K
500
0
G0-G1
84.4
Sub G1
0.33
S
2.08
G2-M
13.2
0
50K
100K
150K
200K
250K
PI-A
EV
Count
1.5K
1.0K
500
0
G0-G1
72.2
Sub G1
0.28
S
2.07
G2-M
24.8
PI-A
TCF19- sh11
Count
1.5K
1.0K
500
0
G0-G1
78.1
Sub G1
0.22
S
3.30
G2-M
18.3
PI-A
TCF19- shUTR

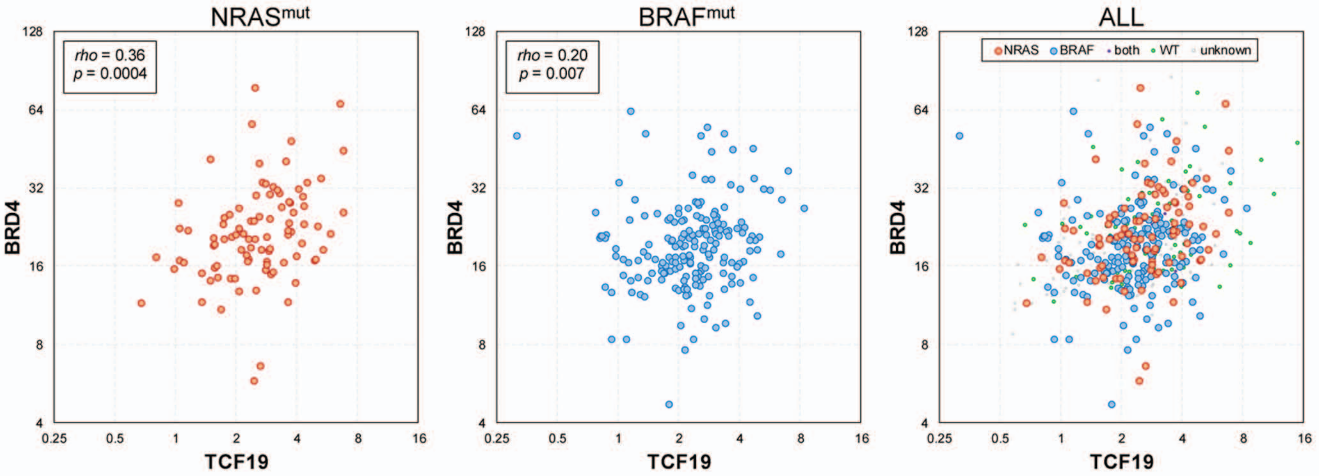

Mutation	n	*rho*	p-value
NRAS	93	0.360	0.00044
BRAF	185	0.200	0.0067
WT/WT	85	0.232	0.033
all	472	0.318	$2x10^{-12}$

S9

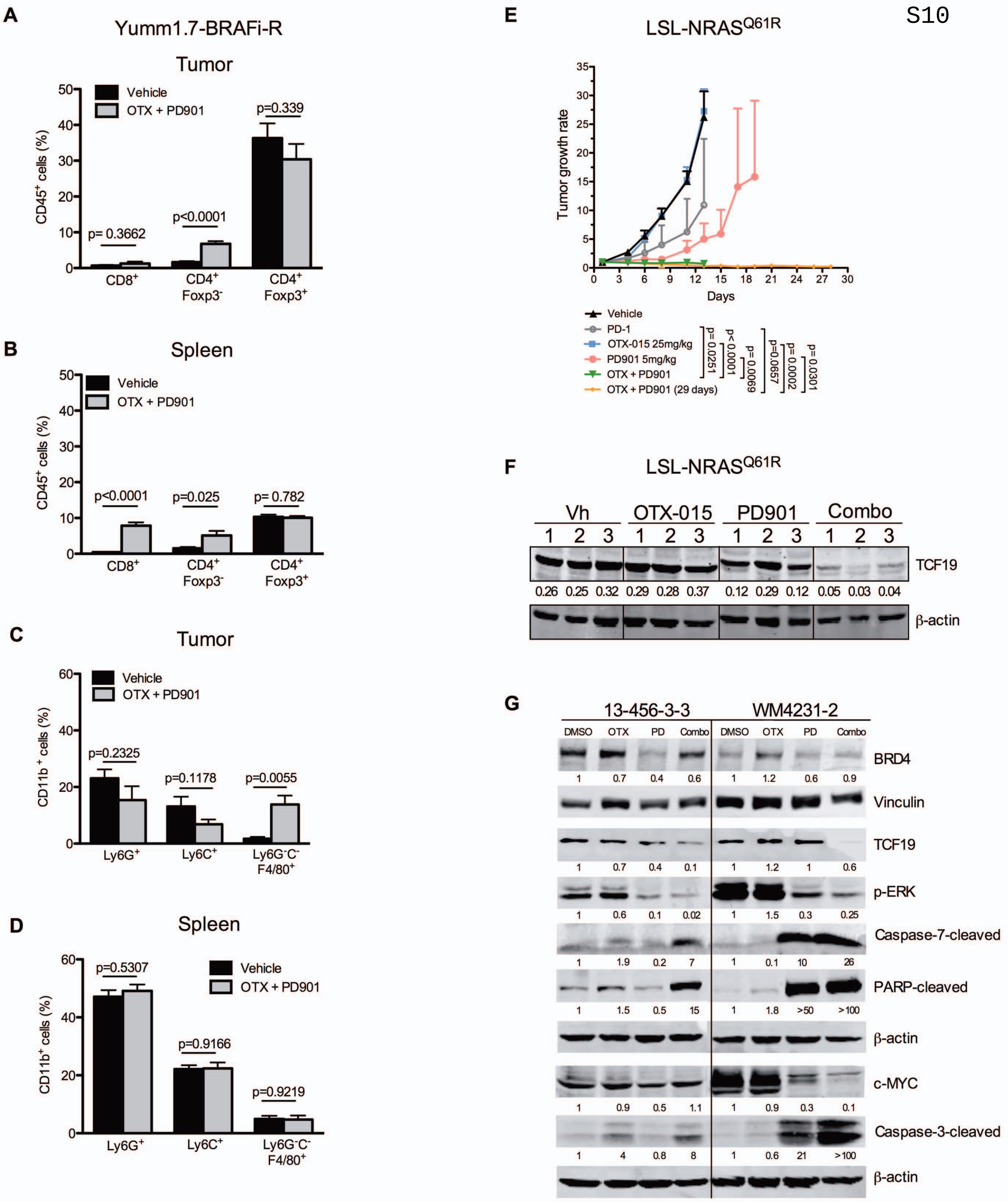
A
Yumm1.7-BRAFi-R
Tumor
Vehicle
OTX + PD901
CD45+ cells (%)
p= 0.3662
p<0.0001
p=0.339
CD8+
CD4+ Foxp3-
CD4+ Foxp3+
B
Spleen
Vehicle
OTX + PD901
CD45+ cells (%)
p<0.0001
p=0.025
p= 0.782
CD8+
CD4+ Foxp3-
CD4+ Foxp3+
C
Tumor
Vehicle
OTX + PD901
CD11b + cells (%)
p=0.2325
p=0.1178
p=0.0055
Ly6G+
Ly6C+
Ly6G-C- F4/80+
D
Spleen
Vehicle
OTX + PD901
CD11b+ cells (%)
p=0.5307
p=0.9166
p=0.9219
Ly6G+
Ly6C+
Ly6G-C- F4/80+
E
LSL-NRASQ61R
Tumor growth rate
Days
Vehicle
PD-1
OTX-015 25mg/kg
PD901 5mg/kg
OTX + PD901
OTX + PD901 (29 days)
p=0.0251
p<0.0001
p=0.0069
p=0.0657
p=0.0002
p=0.0301
F
LSL-NRASQ61R
Vh
OTX-015
PD901
Combo
1 2 3 1 2 3 1 2 3 1 2 3
TCF19
0.26 0.25 0.32 0.29 0.28 0.37 0.12 0.29 0.12 0.05 0.03 0.04
β-actin
G
13-456-3-3
WM4231-2
DMSO OTX PD Combo DMSO OTX PD Combo
BRD4
1 0.7 0.4 0.6 1 1.2 0.6 0.9
Vinculin
TCF19
1 0.7 0.4 0.1 1 1.2 1 0.6
p-ERK
1 0.6 0.1 0.02 1 1.5 0.3 0.25
Caspase-7-cleaved
1 1.9 0.2 7 1 0.1 10 26
PARP-cleaved
1 1.5 0.5 15 1 1.8 >50 >100
β-actin
c-MYC
1 0.9 0.5 1.1 1 0.9 0.3 0.1
Caspase-3-cleaved
1 4 0.8 8 1 0.6 21 >100
β-actin

Supplementary Tables

Appendix Table S1: IC50 values for JQ-1 in a panel of NRAS mutant melanoma and non-transformed cell lines

Appendix Table S2A and S2B: Statistical analysis corresponding to Figure 3C and 3E

Appendix Table S3: P values corresponding to statistical analysis performed for Appendix figure S6

Appendix Table S4A and S4B: Statistical analysis corresponding to Figure 6A and 6C

Appendix Table S5: Information about Melanoma Patient serial tumor biopsies used for RNA- sequencing analysis

Appendix Table S6: Mutation status of short-term cultures derived from immunotherapy resistant patients

Appendix Table S7: List of primers used for real-time RT-PCR analysis

Appendix Table S8: List of antibodies used for this study

Table S1: IC50 values for JQ-1

Cell line	Mutation	Day 3 IC_{50} (μM)
UACC-1273	NRAS-Q61L	0.1516
M93-047	NRAS-Q61L	0.2645
FS13	NRAS-Q61L	0.776
WM852	NRAS-Q61R	1.166
WM3451	NRAS-Q61K	1.366
WM3000	NRAS-Q61K	2.706
WM4113	NRAS-Q61R	29.82
Melanocytes	WT	11.88
Fibroblasts	WT	>30

Table S2A: Statistical analysis corresponding to Figure 3C

Summary tumor growth rate:

Treatment	N	mean	se
Vehicle	3	90.452	15.311
JQ-1 (25mg/kg)	3	92.016	16.142
PD901 (5mg/kg)	3	29.343	0.679
Combo (JQ-1 + PD901)	3	2.916	0.090

Test results:

Comparison	P value
Vehicle vs. JQ-1	0.947338
Vehicle vs. PD901	0.057158
Vehicle vs. Combo	0.029256
JQ-1 vs. Combo	0.031285
PD901 vs. Combo	0.000544

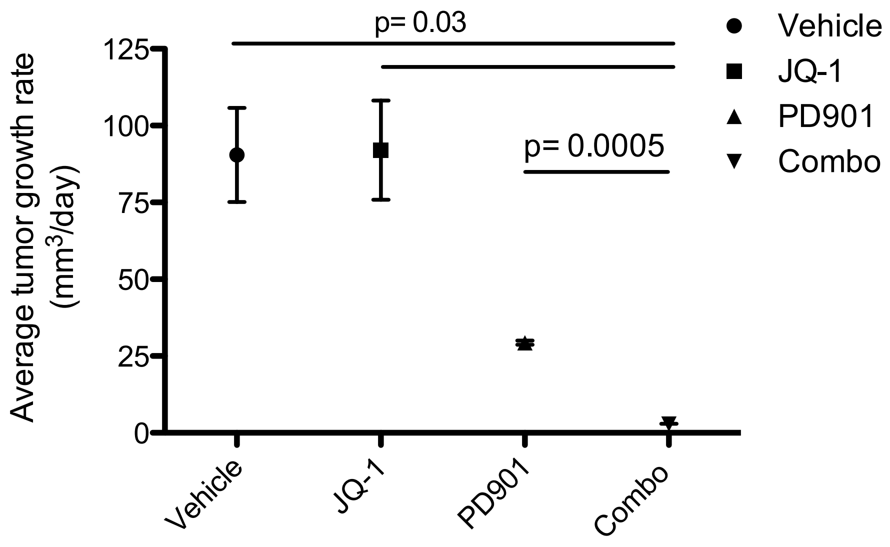

Table S2B: Statistical analysis corresponding to Figure 3E

Summary tumor growth rate:

Treatment	N	mean	se
Vehicle	7	105.304	20.505
OTX-015 (25mg/Kg)	7	111.304	14.870
PD901 (5mg/Kg)	7	46.552	16.112
Combo (OTX-015 + PD901)	7	7.713	2.055

Test results:

Comparison	P value
Vehicle vs. OTX-015	0.817115
Vehicle vs. PD901	0.044922
Vehicle vs. Combo	0.00304
OTX-015 vs. PD901	0.012143
OTX-015 vs. Combo	0.000389
PD901 vs. Combo	0.052632

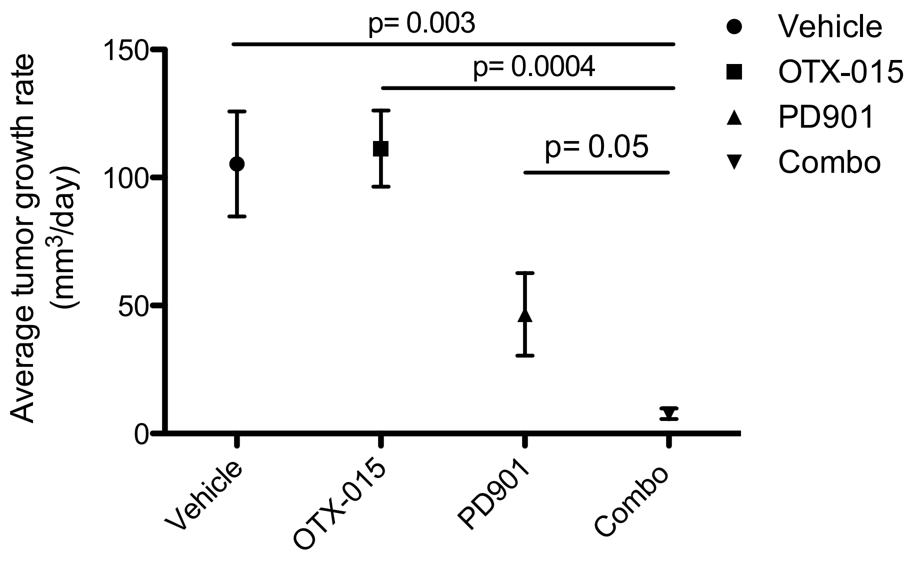

Appendix Table S3: P values corresponding to statistical analysis performed for supplementary figure S6.
Unpaired t test, two tailed

Comparison	vs	p-value
M93-047 β-Actin	M93-047-JQ-ORC1	0.0015
M93-047 β-Actin	M93-047-PD901-ORC1	0.0236
M93-047 β-Actin	M93-047-Combo-ORC1	<0.0001
M93-047 β-Actin	M93-047-JQ-PLK1	<0.0001
M93-047 β-Actin	M93-047-PD901-PLK1	<0.0001
M93-047 β-Actin	M93-047-Combo-PLK1	<0.0001
M93-047 β-Actin	M93-047-JQ-PLK4	<0.0001
M93-047 β-Actin	M93-047-PD901-PLK4	<0.0001
M93-047 β-Actin	M93-047-Combo-PLK4	<0.0001
M93-047 β-Actin	M93-047-JQ-CDK1	<0.0001
M93-047 β-Actin	M93-047-PD901-CDK1	0.0001
M93-047 β-Actin	M93-047-Combo-CDK1	<0.0001
M93-047 β-Actin	M93-047-JQ-AURKA	0.0002
M93-047 β-Actin	M93-047-PD901-AURKA	0.0005
M93-047 β-Actin	M93-047-Combo-AURKA	<0.0001
M93-047 β-Actin	M93-047-JQ-CDT1	0.0002
M93-047 β-Actin	M93-047-PD901-CDT1	0.0019
M93-047 β-Actin	M93-047-Combo-CDT1	<0.0001
M93-047 β-Actin	M93-047-JQ-CDC20	<0.0001
M93-047 β-Actin	M93-047-PD901-CDC20	<0.0001
M93-047 β-Actin	M93-047-Combo-CDC20	<0.0001
M93-047 β-Actin	M93-047-JQ-CDC25C	<0.0001
M93-047 β-Actin	M93-047-PD901-CDC25C	<0.0001
M93-047 β-Actin	M93-047-Combo-CDC25C	<0.0001
M93-047 β-Actin	M93-047-JQ-TOP2A	<0.0001
M93-047 β-Actin	M93-047-PD901-TOP2A	<0.0001
M93-047 β-Actin	M93-047-Combo-TOP2A	<0.0001
M93-047 β-Actin	M93-047-JQ-FANCD2	<0.0001

M93-047 β-Actin	M93-047-PD901-FANCD2	0.0006
M93-047 β-Actin	M93-047-Combo-FANCD2	<0.0001
M93-047 β-Actin	M93-047-JQ-PKMYT1	<0.0001
M93-047 β-Actin	M93-047-PD901-PKMYT1	<0.0001
M93-047 β-Actin	M93-047-Combo-PKMYT1	<0.0001
M93-047 β-Actin	M93-047-JQ-DDIAS	0.9217
M93-047 β-Actin	M93-047-PD901-DDIAS	<0.0001
M93-047 β-Actin	M93-047-Combo-DDIAS	<0.0001
M93-047 β-Actin	M93-047-JQ-BCL2	0.1379
M93-047 β-Actin	M93-047-PD901-BCL2	0.0045
M93-047 β-Actin	M93-047-Combo-BCL2	<0.0001
M93-047 β-Actin	M93-047-JQ-BIRC5	0.0059
M93-047 β-Actin	M93-047-PD901-BIRC5	<0.0001
M93-047 β-Actin	M93-047-Combo-BIRC5	<0.0001
M93-047 β-Actin	M93-047-JQ-BIRC3	0.0941
M93-047 β-Actin	M93-047-PD901-BIRC3	0.0618
M93-047 β-Actin	M93-047-Combo-BIRC3	0.0076

**Comparison**	**vs**	**p-value**
WM3000 β-Actin	WM3000-JQ-ORC1	0.0004
WM3000 β-Actin	WM3000-PD901-ORC1	<0.0001
WM3000 β-Actin	WM3000-Combo-ORC1	<0.0001
WM3000 β-Actin	WM3000-JQ-PLK1	0.0274
WM3000 β-Actin	WM3000-PD901-PLK1	<0.0001
WM3000 β-Actin	WM3000-Combo-PLK1	<0.0001
WM3000 β-Actin	WM3000-JQ-PLK4	0.0345
WM3000 β-Actin	WM3000-PD901-PLK4	0.0009
WM3000 β-Actin	WM3000-Combo-PLK4	<0.0001
WM3000 β-Actin	WM3000-JQ-CDK1	0.211
WM3000 β-Actin	WM3000-PD901-CDK1	<0.0001
WM3000 β-Actin	WM3000-Combo-CDK1	<0.0001
WM3000 β-Actin	WM3000-JQ-AURKA	0.0001

WM3000 β-Actin	WM3000-PD901-AURKA	<0.0001
WM3000 β-Actin	WM3000-Combo-AURKA	<0.0001
WM3000 β-Actin	WM3000-JQ-CDT1	0.0017
WM3000 β-Actin	WM3000-PD901-CDT1	0.0496
WM3000 β-Actin	WM3000-Combo-CDT1	<0.0001
WM3000 β-Actin	WM3000-JQ-CDC20	0.8417
WM3000 β-Actin	WM3000-PD901-CDC20	0.0004
WM3000 β-Actin	WM3000-Combo-CDC20	<0.0001
WM3000 β-Actin	WM3000-JQ-CDC25C	0.0003
WM3000 β-Actin	WM3000-PD901-CDC25C	<0.0001
WM3000 β-Actin	WM3000-Combo-CDC25C	<0.0001
WM3000 β-Actin	WM3000-JQ-TOP2A	0.1558
WM3000 β-Actin	WM3000-PD901-TOP2A	<0.0001
WM3000 β-Actin	WM3000-Combo-TOP2A	<0.0001
WM3000 β-Actin	WM3000-JQ-FANCD2	0.0111
WM3000 β-Actin	WM3000-PD901-FANCD2	0.0003
WM3000 β-Actin	WM3000-Combo-FANCD2	0.0012
WM3000 β-Actin	WM3000-JQ-PKMYT1	0.2572
WM3000 β-Actin	WM3000-PD901-PKMYT1	<0.0001
WM3000 β-Actin	WM3000-Combo-PKMYT1	<0.0001
WM3000 β-Actin	WM3000-JQ-DDIAS	0.857
WM3000 β-Actin	WM3000-PD901-DDIAS	0.0004
WM3000 β-Actin	WM3000-Combo-DDIAS	<0.0001
WM3000 β-Actin	WM3000-JQ-BCL2	0.0402
WM3000 β-Actin	WM3000-PD901-BCL2	0.0011
WM3000 β-Actin	WM3000-Combo-BCL2	0.0005
WM3000 β-Actin	WM3000-JQ-BIRC5	0.562
WM3000 β-Actin	WM3000-PD901-BIRC5	0.0021
WM3000 β-Actin	WM3000-Combo-BIRC5	<0.0001
WM3000 β-Actin	WM3000-JQ-BIRC3	0.0216
WM3000 β-Actin	WM3000-PD901-BIRC3	0.0518
WM3000 β-Actin	WM3000-Combo-BIRC3	0.4661

Comparison	vs	p-value
M93-047 β-Actin	M93-047-JQ-TCF19	0.0903
M93-047 β-Actin	M93-047-PD901-TCF19	0.0053
M93-047 β-Actin	M93-047-Combo-TCF19	<0.0001
M93-047 β-Actin	M93-047-JQ-E2F1	0.2873
M93-047 β-Actin	M93-047-PD901-E2F1	0.0011
M93-047 β-Actin	M93-047-Combo-E2F1	<0.0001
M93-047 β-Actin	M93-047-JQ-E2F3	0.2803
M93-047 β-Actin	M93-047-PD901-E2F3	0.0055
M93-047 β-Actin	M93-047-Combo-E2F3	0.4185
WM3000 β-Actin	WM3000-JQ-TCF19	0.0421
WM3000 β-Actin	WM3000-PD901-TCF19	0.0001
WM3000 β-Actin	WM3000-Combo-TCF19	<0.0001
WM3000 β-Actin	WM3000-JQ-E2F1	0.006
WM3000 β-Actin	WM3000-PD901-E2F1	<0.0001
WM3000 β-Actin	WM3000-Combo-E2F1	<0.0001
WM3000 β-Actin	WM3000-JQ-E2F3	0.0068
WM3000 β-Actin	WM3000-PD901-E2F3	0.1203
WM3000 β-Actin	WM3000-Combo-E2F3	0.0004
WM852 β-Actin	WM852-JQ-TCF19	0.6073
WM852 β-Actin	WM852-PD901-TCF19	0.0516
WM852 β-Actin	WM852-Combo-TCF19	0.0103
WM852 β-Actin	WM852-JQ-E2F1	0.5528
WM852 β-Actin	WM852-PD901-E2F1	0.9884
WM852 β-Actin	WM852-Combo-E2F1	0.0028
WM852 β-Actin	WM852-JQ-E2F3	0.5218
WM852 β-Actin	WM852-PD901-E2F3	0.0376
WM852 β-Actin	WM852-Combo-E2F3	0.3498

Appendix Table S4A: Statistical analysis corresponding to Figure 6A

Summary tumor growth rate:

Treatment	N	mean	se
Vehicle	8	266.344	37.716
PD901 (5mg/Kg)	8	98.104	24.555
OTX-015 (25mg/Kg)	8	285.651	60.983
Combo	15	34.264	7.299

Test results:

Comparison	P value
Vehicle vs. PD901	0.002819
Vehicle vs. OTX-015	0.792423
Vehicle vs. Combo	0.000392
PD901 vs. OTX-015	0.018574
PD901 vs. Combo	0.036517
OTX-015 vs. Combo	0.004343

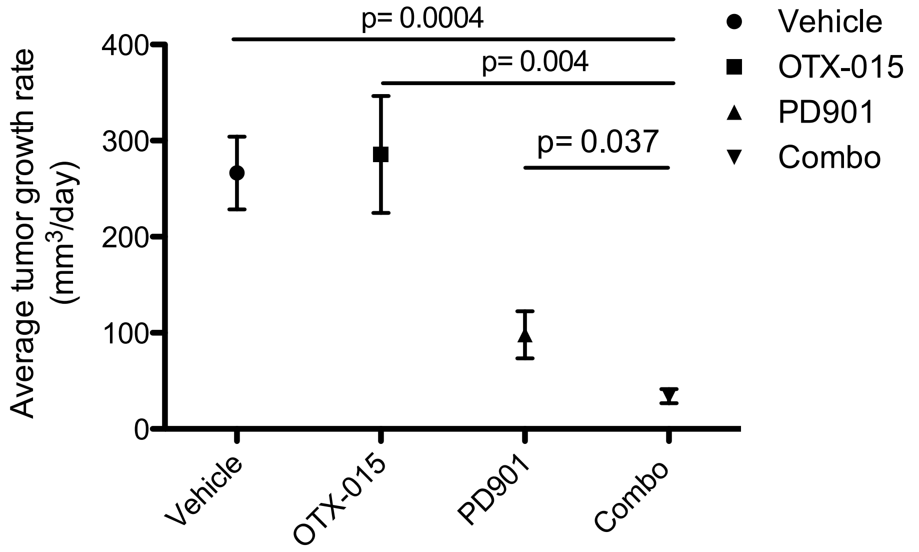

Appendix Table S4B: Statistical analysis corresponding to Figure 6C

Summary tumor growth rate:

Treatment	N	mean	se
Vehicle	7	146.786	20.444
OTX-015 (25mg/kg)	7	155.841	23.002
PD901 (5mg/kg)	7	50.861	6.122
Combo (OTX+PD901)	7	19.522	9.882

Test results:

Comparison	P value
Vehicle vs. OTX-015	0.773674
Vehicle vs. PD901	0.002749
Vehicle vs. Combo	0.000382
OTX-015 vs. PD901	0.00329
OTX-015 vs. Combo	0.000577
PD901 vs. Combo	0.022441

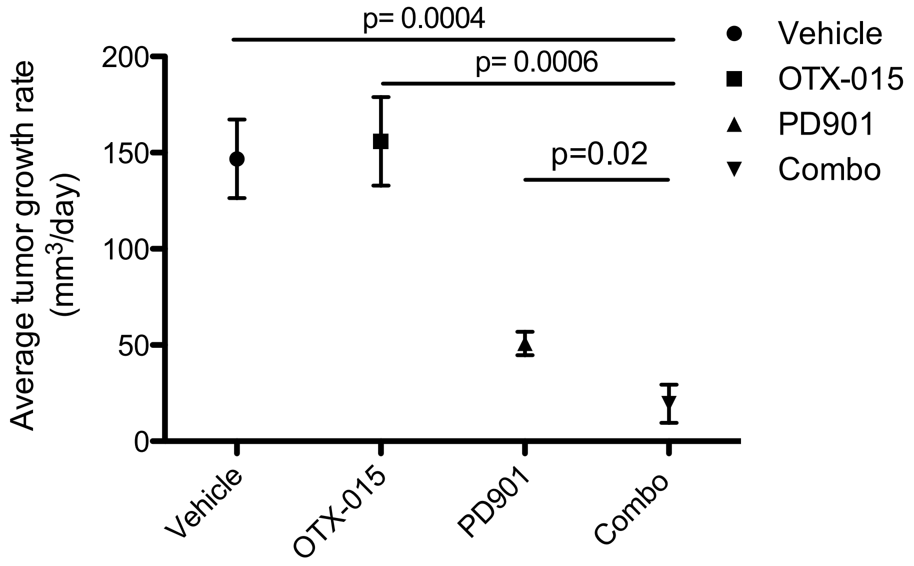

Appendix Table S5: Information about serial tumor biopsies from melanoma patients used for RNA-sequencing analysis.

PATIENT	TREATMENT	SAMPLE	CLINICAL RESPONSE	RESPONSE	TCF19 NORMALIZED VALUES (FPMK)
1	pre-PD-1-combo	Baseline			0.14
	post-PD-1-combo (209 days)	Progression	Progressive Disease	NR	0.19
2	pre-CTLA-4; pre-PD-1	Baseline			0.07
	post-CTLA-4 (235 days); on-PD-1 (3 days)	Progression	Progressive Disease	NR	0.08
3	post-BRAFi (393 days); post-IL2 (388 days); post-IPI (153 days); pre-dab+tra; pre-Pembro	Baseline			0.11
	post-BRAFi (540 days); post-IL2 (535 days); post-IPI (300 days); post-dab+tra (54 days); on-Pembro (21 days)	On-treatment	Progressive Disease	NR	0.13
	post-BRAFi (607 days); post-IL2 (602 days); post-IPI (367 days); post-dab+tra (121 days); on-Pembro (88 days)	On-treatment	Progressive Disease	NR	0.13
4	post-BRAFi (479 days); pre-Nivo	Baseline			0.00
	post-BRAFi (960 days); post-Nivo (31 days)	Progression	Progressive Disease	NR	0.00
5	post-LGX+MEK; post-IPI (176 days); post-dab+tra (64 days); pre-Pembro	Baseline			1.28
	post-LGX+MEK; post-IPI (214 days); post-dab+tra (102 days); post-Pembro (38 days)	Progression	Progressive Disease	NR	2.07
	post-LGX+MEK; post-IPI (250 days); post-dab+tra (138 days); post-Pembro (74 days)	Progression	Progressive Disease	NR	1.99
	post-LGX+MEK; post-IPI (250 days); post-dab+tra (138 days); post-Pembro (74 days)	Progression	Progressive Disease	NR	2.25
6		Baseline			6.18
	BRAFi + MEKi	On-treatment	Stable Disease	R	3.26
7		Baseline			8.48
	BRAFi + MEKi	On-treatment	Partial Response	R	3.32
8		Baseline			11.33
	BRAFi + MEKi	On-treatment	Partial Response	R	5.77
9		Baseline			3.34
	BRAFi	Progression	Progressive Disease	NR	9.13
10		Baseline,			13.90
	BRAFi + MEKi	On-treatment	Stable Disease	R	7.63
11		Baseline			3.56
	BRAFi + MEKi	On-treatment	Partial Response	R	3.18
12		Baseline			10.81
	BRAFi + MEKi	Progression	Progressive Disease	NR	6.42
13		Baseline			10.49
	BRAFi	On-treatment	Partial Response	R	3.98
14		Baseline			9.18
	BRAFi + MEKi	Progression	Progressive Disease	NR	8.10
15		Baseline			9.85
	BRAFi + MEKi	On-treatment	Partial Response	R	8.49
16		Baseline			9.92
	BRAFi + MEKi	On-treatment	Complete Response	R	5.45
17	pre-IPI; pre-PD1	Baseline			4.39
	post-IPI (22 days); pre-PD1	On-treatment	Partial Response	R	4.55
18	pre-IPI	Baseline			3.91
	post-IPI (47 days)	On-treatment	Partial Response	R	3.25
19	post-PD1 (83 days); pre-IPI	Baseline			4.28
	post-PD1 (109 days); post-IPI (25 days)	On-treatment	Stable Disease	R	2.71
	post-PD1 (182 days); post-IPI (98 days)	On-treatment	Partial Response	R	1.70
20		Baseline		R	6.25
	BRAFi	On-treatment	Partial Response		4.73
21	vem+cobi (prior IFN)	Baseline			0.69
	vem+cobi (prior IFN)	On-treatment	Stable Disease	R	0.37
22	IFN (pre-PDL1)	Baseline			0.40
	PDL1	On-treatment	Partial Response	R	0.16
23	Pembro	Baseline			0.38
	Pembro	On-treatment	Progressive Disease	NR	0.53

- Patients who achieved clinical benefit (Complete response, partial response or stable disease) at the time of biopsy were considered responders
- Patients who did not achieved clinical benefit (progressive disease) were considered non-responders
- Samples were obtained from 23 patients. For some patients multiple samples taken at different times were available.
- Samples from patient 4 were not considered, as TCF19 levels were not detected.
- Samples were obtained before treatment initiation (Baseline), during treatment (On-treatment) or at relapse (Progression)
- BRAFi: BRAF inhibitor; LGX: LGX818 (Encorafenib); dab: dabrafenib
- MEKi: MEK inhibitor; tra: trametinib; cobi: cobimetinib (GDC-0973, RG7420)
- Ipi: Ipilimumab (anti-CTLA4 antibody)
- Pembro: Pembrolizumab (anti-PD1 antibody)
- PDL1 (anti-PDL1 antibody)

Appendix Table S6: Mutation status of the short-term cultures derived from immunotherapy resistant patients

Short-term primary culture	Disease type	Mutation	Therapy resistance
13-456-3-3	Human melanoma	*BRAF*^{V600E}	Anti-CTLA4<br>Anti-PD1
13-456-5-3	Human melanoma	*BRAF*^{V600E}	Radiation<br>Anti-CTLA4<br>Anti-PD1
15-1761-1-2	Human melanoma	*NRAS*^{Q61R}<br>*BRAF*^{F467L}	Anti-PD1
WM4231-2	Human melanoma	*NRAS*^{Q61R}<br>*BRAF*^{V624R}	Rad Vax<br>Anti-CTLA4
WM4265-1	Human melanoma	*NRAS*^{Q61R}	Anti-CTLA4<br>Anti-PD1

Appendix Table S7. List of Primers used for real-time RT-PCR analysis.

Gene	Forward (5'->3')	Reverse (5'->3')
E2F3	AGGAGCTTTGTCCCATCGTG	GCTCCTTGGGGCTGTTACAT
E2F2	GAGCTCACTCAGACCCCAAG	AACAGGCTGAAGCCAAAAGA
E2F1	CCGGGGAATGAAGGTGAACA	GAGCAAAAGGGCCGAAAGTG
TCF19	TGACGTCTGGTTCCATGTGG	CCTTGGCAGTGGACCTTAGG
PLK1	TGACTCAACACGCCTCATCC	GCTCGCTCATGTAATTGCGG
PLK4	CGGAAGGTGTCAGGGAGAAC	GATCTTCTCCCCGATGCAGG
CDC20	GTTCGGGTAGCAGAACACCA	CCCCTTGATGCTGGGTGAAT
TOP2A	GCAGCCCATTGGTCAGTTTG	CATTCAGGCTCAACACGCTG
ORC1	TGAGAACCCGAATTGCAGCT	ATGGGGAGTAGAGGTCGCTT
FANCD2	TCTGGCACTGATGGTTGCAT	GGAGGGAATGGAAATGGGCA
CDC25C	ACTGAGTTGCTGAGGTGTCG	GCCTCTTTCTGCTCAGGGTT
PKMYT1	AGCGGATGTGTTCAGTCTGG	GAAGACAGACCGGCAGTGAA
CDT1	ATGCGTAGGCGTTTTGAGGA	GCTCGATGGTGAGCTGGTAA
AURKA	TACCATTGACTGCTGCCCTC	GTCGAACCTTGCCTCCAGAT
CDK1	AGGCGAAGATCAACATGGCA	CCAATGTCCCCAAGAGCTGT
DDIAS	GCACTAGTGGCTTGCCAGAT	GGTGCCTGAGAGTCACACTG
BCL2	GGGGTCATGTGTGTGGAGAG	CATCCCAGCCTCCGTTATCC
BIRC5	AGTCCCTGGCTCCTCTACTG	TGAAGTTCACCCCGTTTCCC
BIRC3	AAGCTACCTCTCAGCCTACTTT	CCACTGTTTTCTGTACCCGGA
β-actin	AGAGCTACGAGCTGCCTGAC	AGCACTGTGTTGGCGTACAG

Appendix Table S8: Antibodies validation

Antibody	Validation	Reference	Assay
anti-BRD2	Bethyl Cat#A302-582A, RRID:AB_2034828		WB
anti-BRD3	Bethyl Cat#A302-368A, RRID:AB_1907251		WB
anti-BRD4	Bethyl Cat#A301-985A100, RRID:AB_2620184		IHC
anti-Cyclin D1	Millipore Cat#04-1151, RRID:AB_10615820		WB
anti-MYC	Cell Signaling Technology, Inc - 5605		WB
anti-caspase 7 cleaved	Cell Signaling Technology Cat#9491, RRID:AB_2068144		WB
anti-caspase 3 cleaved	Cell Signaling Technology Cat#9661, RRID:AB_2341188		WB
anti-pRB S807/811	Cell Signaling Technology Cat#9308, RRID:AB_331472		WB
anti-PLK1	Cell Signaling Technology Cat#4535, RRID:AB_2252687		WB
anti-AuroraK B	Cell Signaling Technology Cat#3094, RRID:AB_2061777		WB
anti-phospho ERK1/2	Cell Signaling Technology Cat#4370, RRID:AB_2315112		WB
anti-cleaved PARP	https://www.ncbi.nlm.nih.gov/pubmed/29107960		WB

anti-BIRC5	Novus Cat#NB500-201, RRID:AB_10001517		WB
anti-BIM	https://www.ncbi.nlm.nih.gov/pubmed/28445931?dopt=Abstract		WB
anti-TCF19	https://cdn.origene.com/datasheet/ta333897.pdf		WB
anti-β Actin	Sigma-Aldrich Cat#A5441, RRID:AB_476744		WB
APC anti-mouse CD45	https://www.biolegend.com/en-us/products/apc-anti-mouse-cd45-antibody-97	PMID: 22547694	FC
APC/CY7 anti-mouse CD11C	https://www.biolegend.com/en-us/products/apc-cy7-anti-mouse-cd11c-antibody-3931	PMID: 25911761	FC
Alexa flour 700 anti-mouse Ly-	https://www.biolegend.com/en-us/products/alexa-fluor-700-anti-mouse-ly-6g-antibody-6754	PMID: 25452586	FC
Alexa flour 700 anti-mouse	https://www.biolegend.com/en-us/products/alexa-fluor-700-anti-mouse-cd3-antibody-3375	PMID: 25476696	FC
PE anti-mouse CD8a	https://www.biolegend.com/en-us/products/pe-anti-mouse-cd8a-antibody-155	PMID: 25824821	FC
Brilliant violet 510 anti-mouse	https://www.biolegend.com/en-us/products/brilliant-violet-510-anti-mouse-cd4-antibody-7991	PMID: 24829411	FC
PE/Dazzle 594 anti-mouse	https://www.biolegend.com/en-us/products/pe-dazzle-594-anti-mouse-cd152-antibody-10455	PMID: 20538992	FC
PerCp/Cy5 .5 anti-mouse/hu	https://www.biolegend.com/en-us/products/percp-cy5-5-anti-mouse-human-cd45r-b220-antibody-4267	PMID: 20512127	FC
anti-mouse CD11b PE-cyanine 7	https://www.thermofisher.com/order/genome-database/generatePdf?productName=CD11b&assayType=PRANT&detailed=true&productId=25-	PMID: 28199841	FC
Pacific Blue anti-mouse	https://www.biolegend.com/en-us/products/pacific-blue-anti-mouse-foxp3-antibody-4663	PMID: 20335532	FC

anti-mouse F4/80 antigen PE	https://www.thermofisher.com/order/genome-database/generatePdf?productName=F4/80&assayType=PRANT&detailed=true&productId=12-	PMID: 28351984	FC
FITC rat anti-mouse Ly-6C	http://www.bdbiosciences.com/us/reagents/research/antibodies-buffers/immunology-reagents/anti-mouse-antibodies/cell-surface-antigens/fitc-rat-anti-mouse-ly-6c-al-21/p/553104	PMID: 9647212	FC
APC Annexin V	https://www.biolegend.com/en-us/products/apc-annexin-v-8144	PMID: 24759703	FC

WB: Western blot
IHC: Immunohistochemistry
FC: Flow cytometry
