# Supplementary material for: Co‐targeting BET and MEK as salvage therapy for MAPK and checkpoint inhibitor‐resistant melanoma
Source: EMBO Mol Med. 2018 Apr 11;10(5):e8446. doi: 10.15252/emmm.201708446 (PMC5938620; doi:10.15252/emmm.201708446)
Supplement: Supplementary file 2 — Expanded View Figures PDF [file EMMM-10-e8446-s002.pdf]

Expanded View Figures

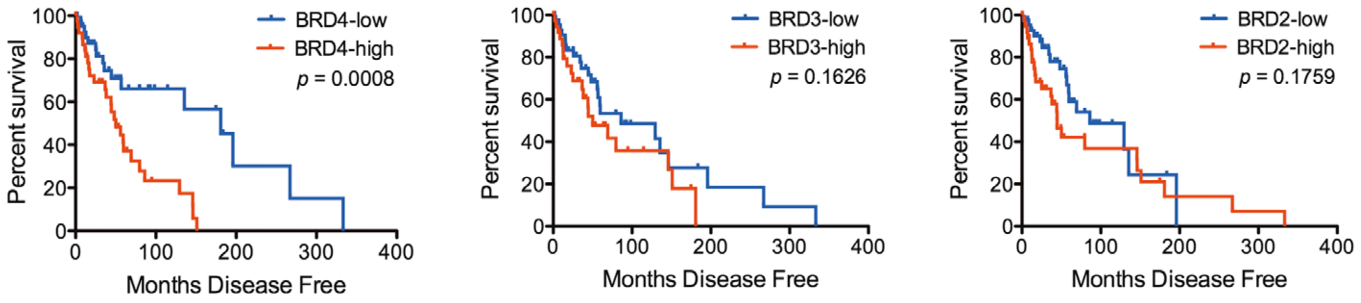

Figure EV1. BRD4 expression levels correlate with disease-free survival in NRAS-mutant melanoma patients.

For all associations of gene expression with survival, patients were split into two groups of high and low BET/BRD expression based on the gene's median expression level. Disease-free survival Kaplan–Meier curves for BRD4, BRD3, BRD2 in the NRAS-mutant group are shown; *P*-values were calculated by long-rank test comparing the two Kaplan–Meier curves.

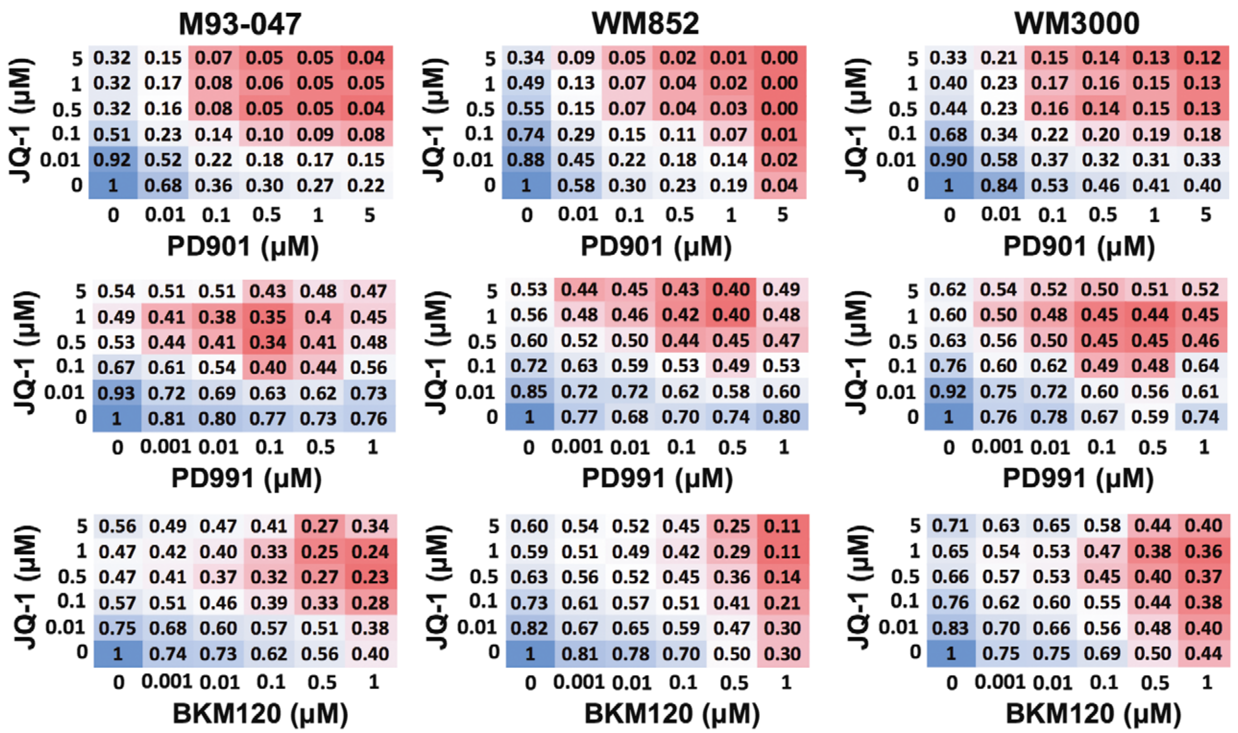

Figure EV2. Sensitivity to combinations of JQ-1 with inhibitors of RAS effector pathways in NRAS-mutant melanoma.

A panel of NRAS-mutant melanoma cells was treated with the indicated doses of JQ1 alone or in combination with the MEKi PD0325901, Cdk4/6i PD0332991, or PI3Ki BKM120 for 5 days. Cell viability was determined by Alamar Blue assay after 5 days of treatment. Relative cell viability (normalized to vehicle-treated cells) is shown for each combination at the indicated doses.

© 2018 The Authors

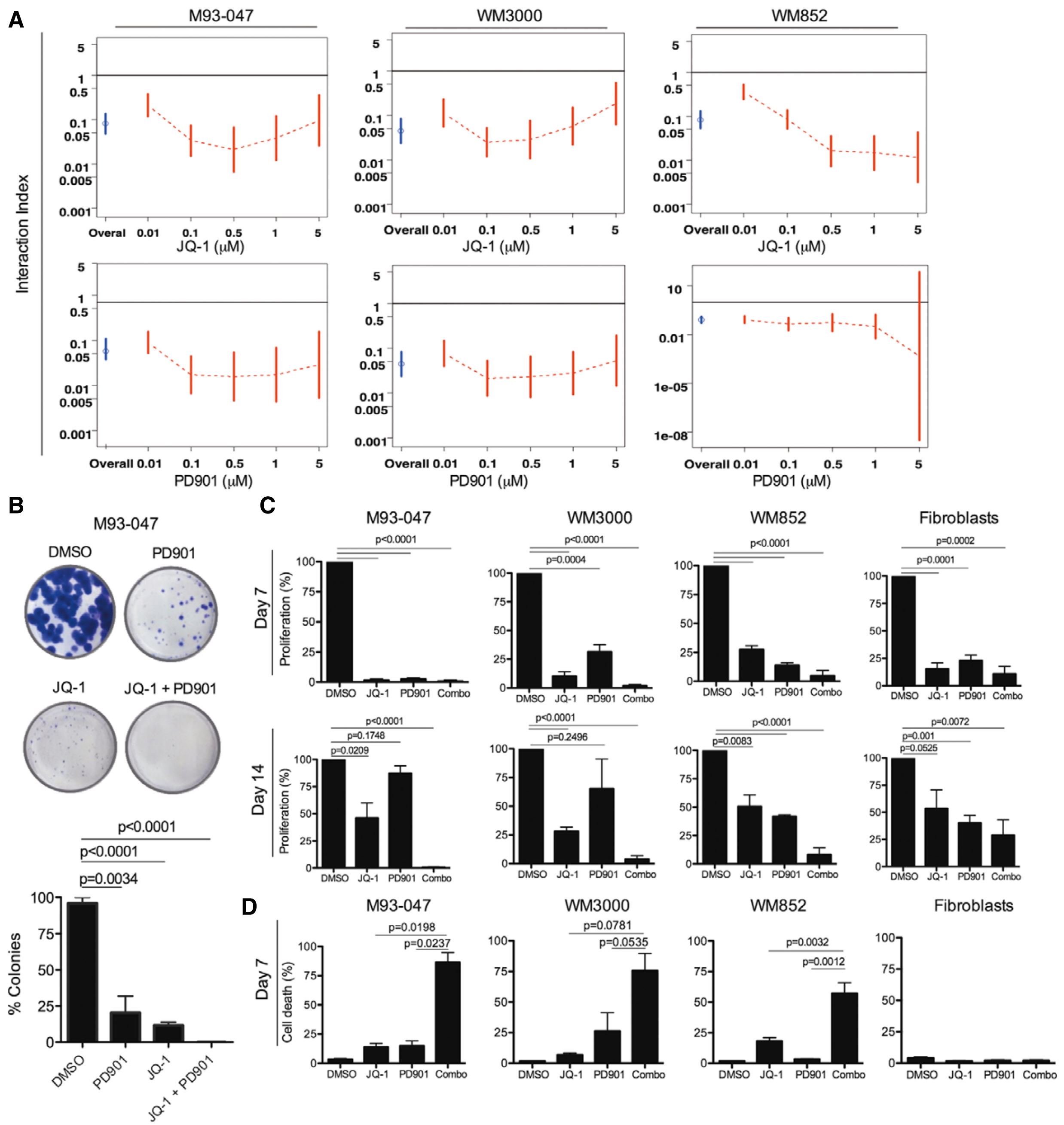

Figure EV3. The BET inhibitor JQ-1 in combination with the MEK inhibitor PD0325901 synergistically impairs cell proliferation and induces apoptosis of NRAS-mutant melanoma cells.

A NRAS-mutant melanoma cells were treated with JQ-1 alone or in combination with PD901. Cell viability was determined by Alamar Blue assay after 5 days of treatment and calculated relative to DMSO-treated controls. Interaction index and 95% confidence interval (CI) were assessed for each cell line. The upper limit of its 95% CI < 1 was considered significant synergy.

B Cells were cultured in the presence of DMSO, 0.5 μM JQ-1, 0.1 μM PD901, or combo for 14 days followed by crystal violet staining. Colonies were imaged using a digital camera and quantitated by ImageJ. Representative photographs of crystal violet stained colonies are shown.

C Cells were treated with DMSO or a single dose of 0.5 μM JQ-1, 0.1 μM PD901, or combo. At day 6, cells were washed to remove the drugs and refed fresh (drug-free) medium. Cells were fixed after 7 or 14 days, stained with crystal violet, and relative number of cells quantified.

D NRAS-mutant melanoma and non-transformed cells were treated as in (C) for 7 days. Cells were stained with propidium iodide and Annexin V-FITC and analyzed by FACS; % Annexin V^+/PI^+ cells are shown.

Data information: Data represent the mean of three independent experiments ± SEM. Statistically significant differences were determined by Student's *t*-test; *P*-values are shown.

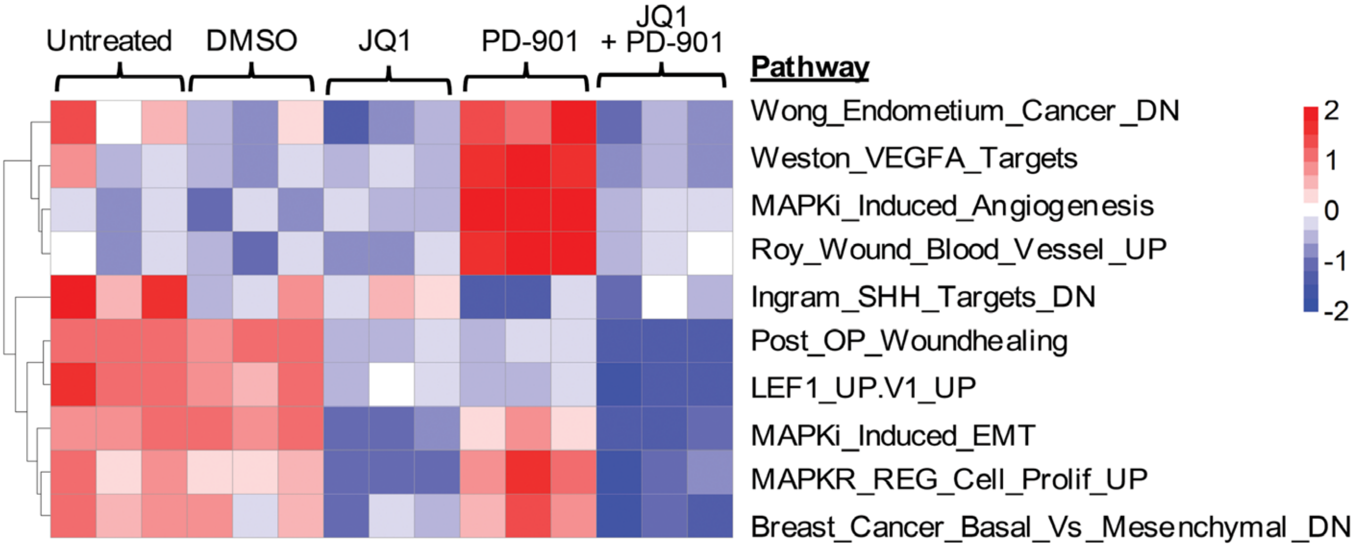

Figure EV4. Co-targeting BET and MEK inhibits innate anti-PD1 resistance (IPRES) gene signatures.

Heat map representation of enrichment scores of 10 innate anti-PD1 resistance (IPRES) gene signatures is shown in M93-047 cells after treatment with DMSO, 0.5 μM JQ-1, 0.1 μM PD901, or combination (JQ-1/PD901) for 48 h.

© 2018 The Authors

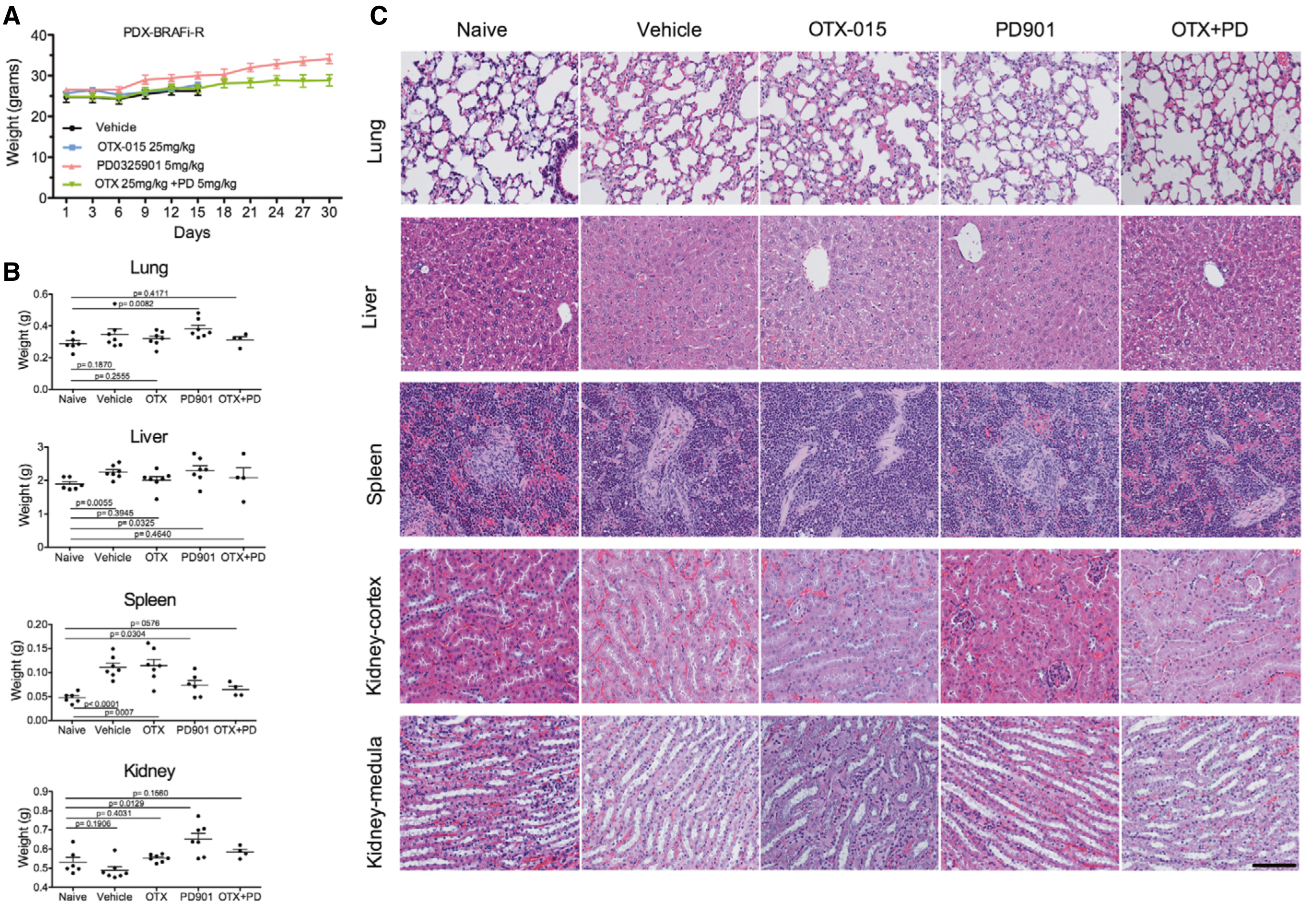

Figure EV5. Combination of OTX-015 and PD0325901 does not induce overt toxicity.

A Weight of mice included in the study. Mice were treated with vehicle, OTX-015 (25 mg/kg po.qd), PD901 (5 mg/kg po.qd), or combination for 30 days.

B Weight of organs from mice included in the study.

C H&E of the major visceral organs of PDX mice. Magnified (20×) images are shown. Scale bar = 100 μm.

Data information: Data represent average weight of seven mice in the Vehicle, OTX-015 and PD901 groups, and four mice in the combination group ± SEM. Statistically significant differences were determined by Student's *t*-test; *P*-values are shown.
