# Supplementary figures and images for: Co‐targeting BET and MEK as salvage therapy for MAPK and checkpoint inhibitor‐resistant melanoma
Source: EMBO Mol Med. 2018 Apr 11;10(5):e8446. doi: 10.15252/emmm.201708446 (PMC5938620; doi:10.15252/emmm.201708446)

Appendix Figure 6D

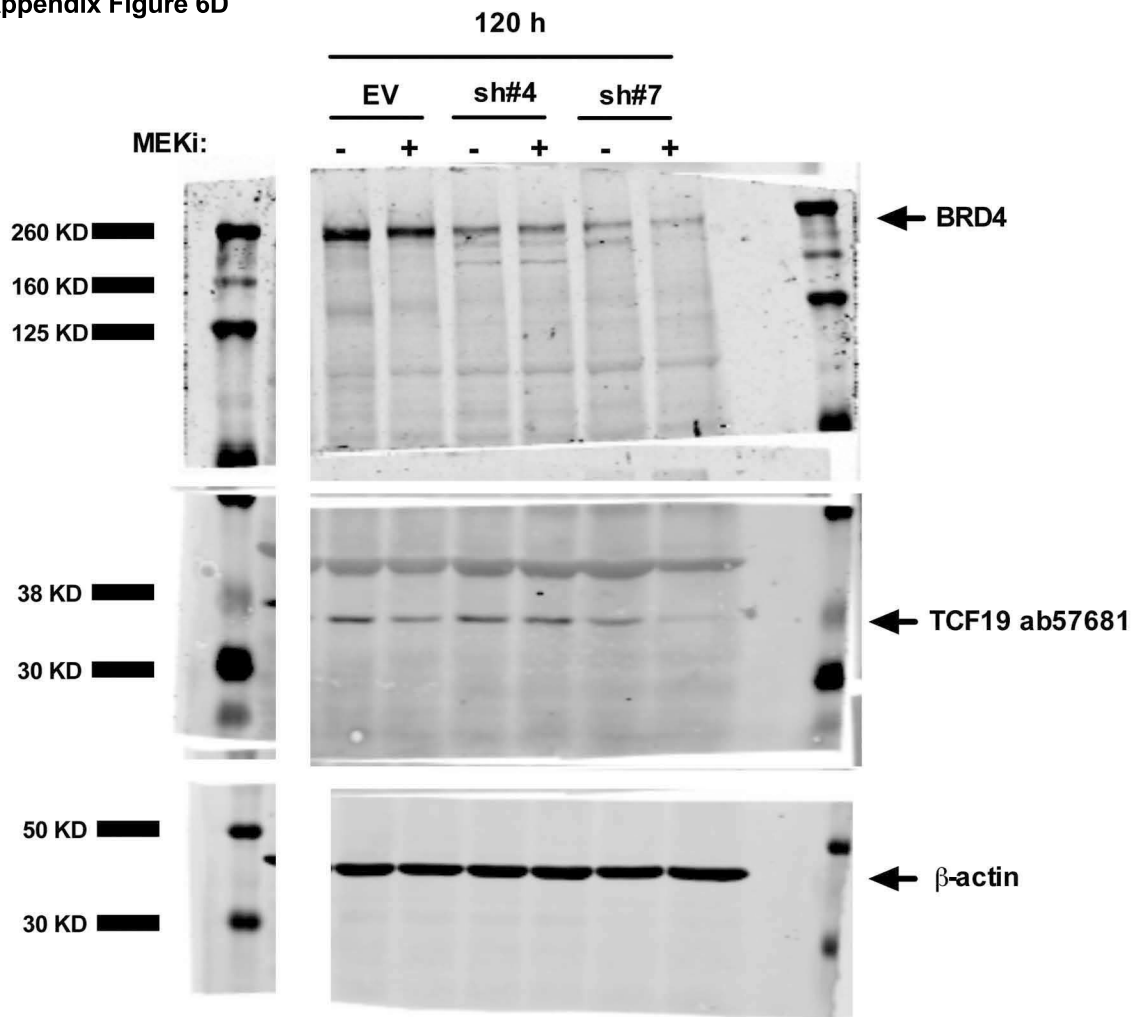

Appendix Figure S6E

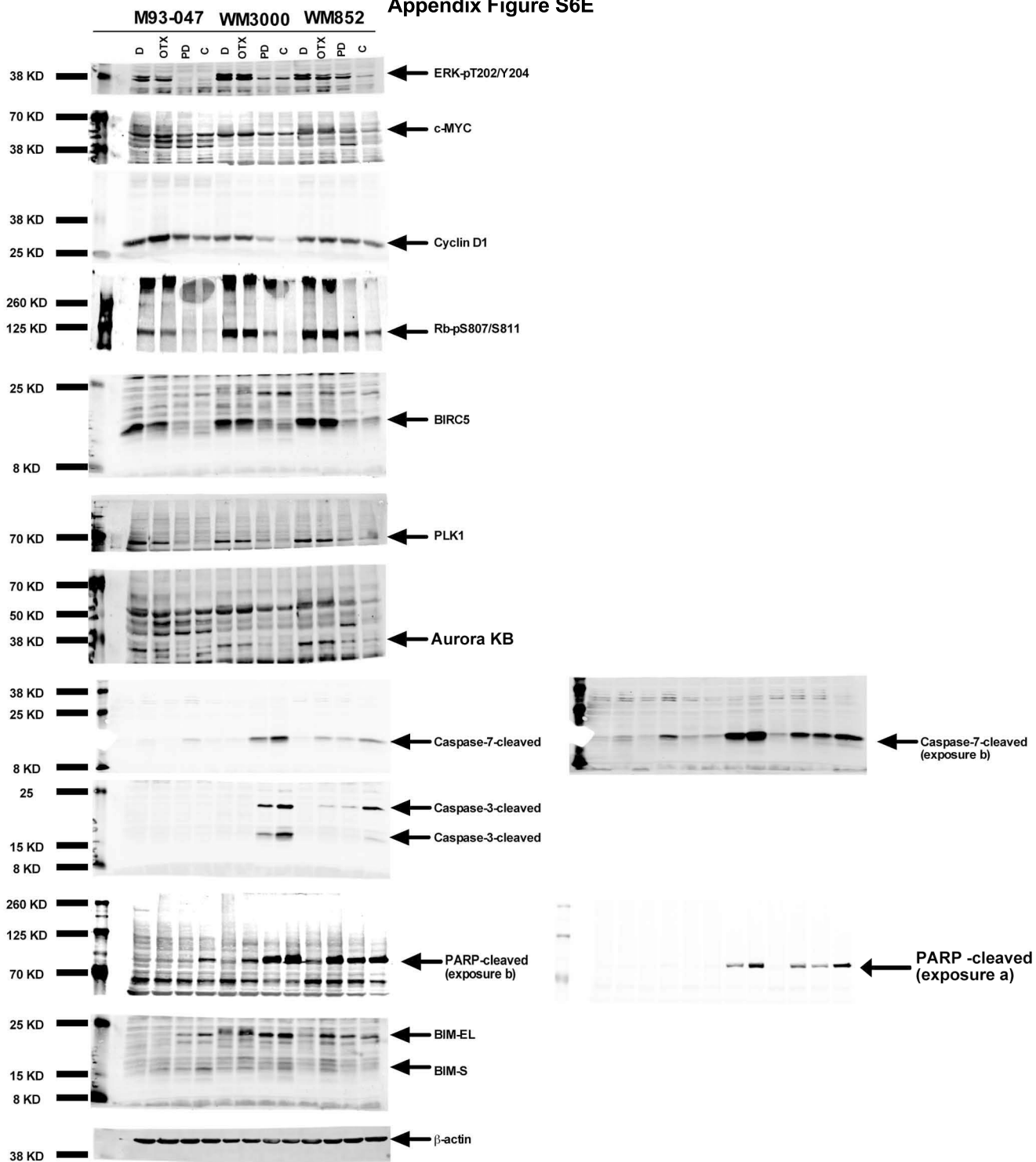

Supplement: Supplementary file 4 — Source Data for Appendix [file EMMM-10-e8446-s010.zip › EMM_08446_Appendix_SD/Appendix_Figure_S6_source_data.pdf]

**Appendix Figure S2A**

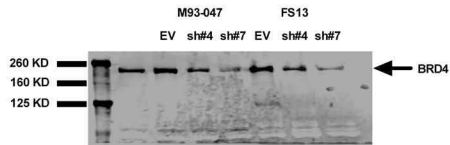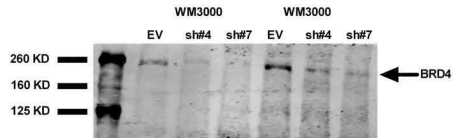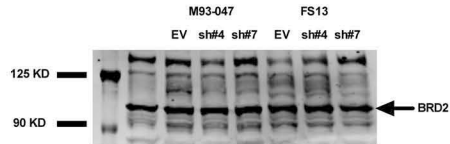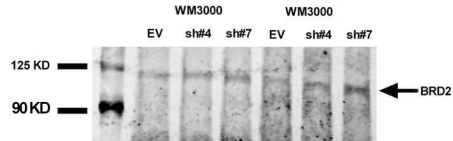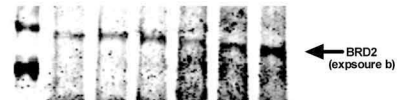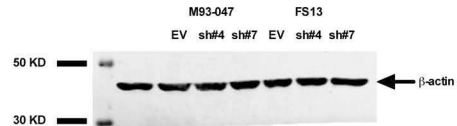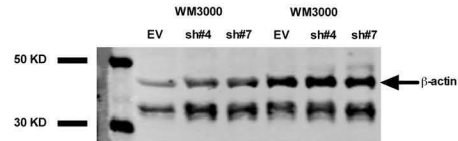

Supplement: Supplementary file 4 — Source Data for Appendix [file EMMM-10-e8446-s010.zip › EMM_08446_Appendix_SD/WB_FigS2A.pdf]

# WHN89 in vivo study Mice Tx for 13-15 days

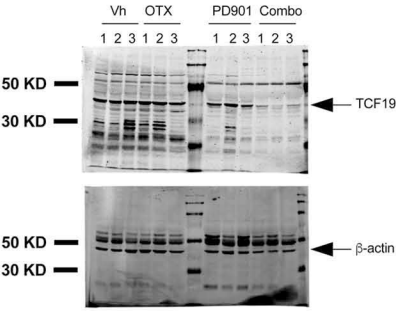

Vh: 1564, 1566, 1550  
OTX: 298, 299, 290  
PD901: 218, 272, 221  
Combo: 263, 1567, 262

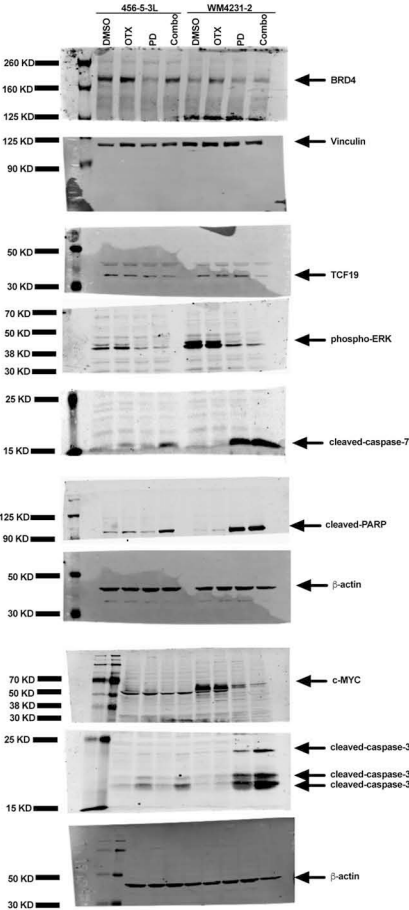

48h Tx

Supplement: Supplementary file 4 — Source Data for Appendix [file EMMM-10-e8446-s010.zip › EMM_08446_Appendix_SD/WB_FigS9F_G.pdf]

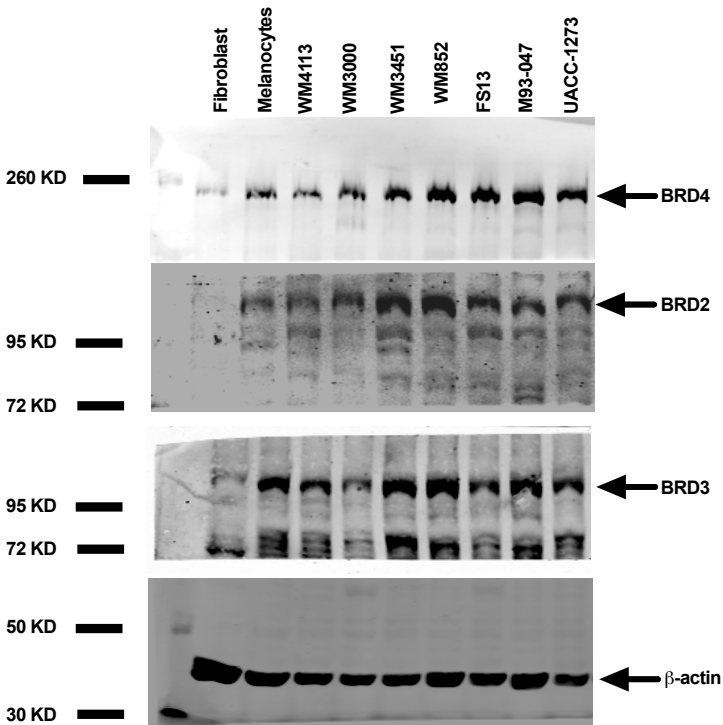

Supplement: Supplementary file 7 — Source Data for Figure 2 [file EMMM-10-e8446-s005.pdf]

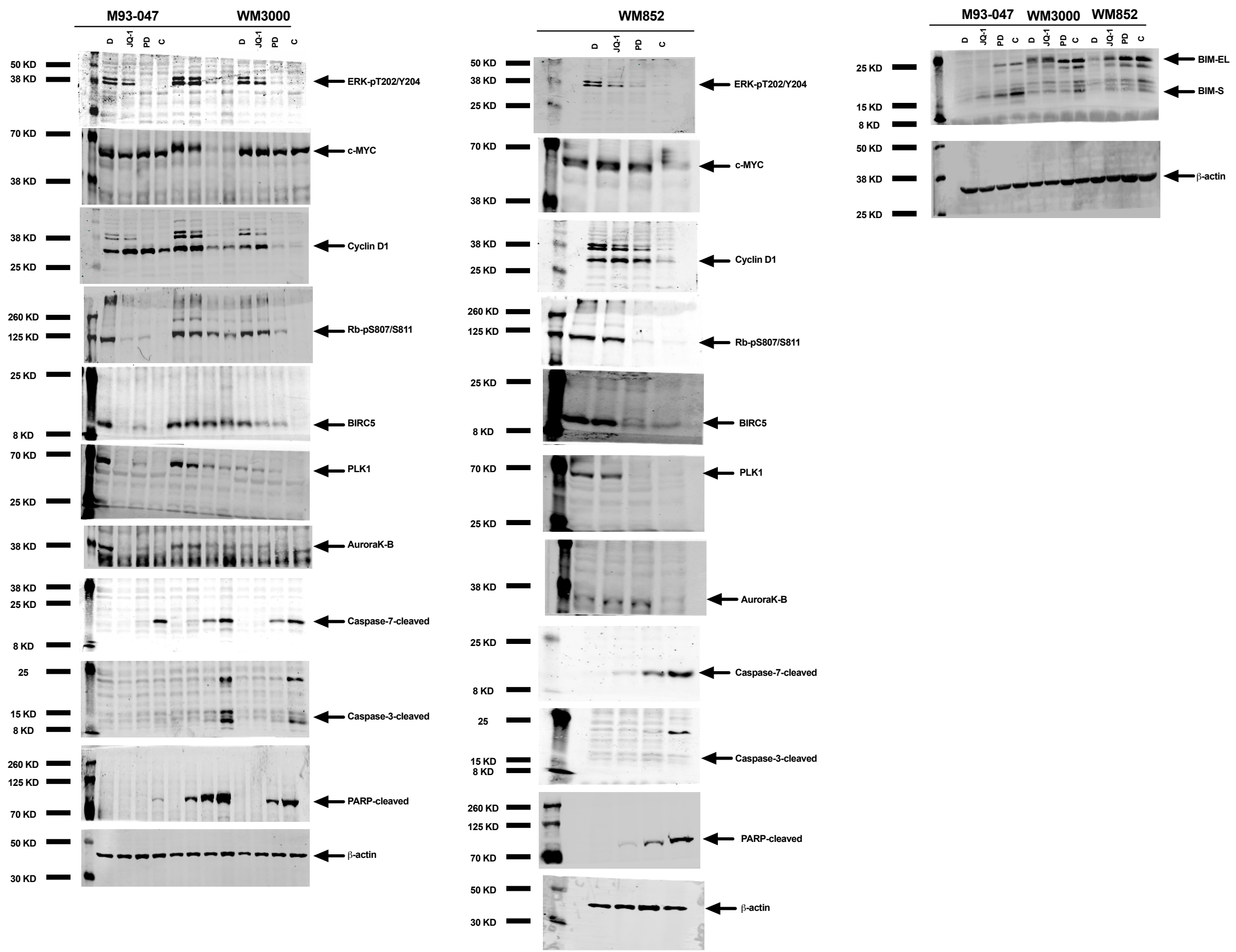

Figshare.com

Figure 4, panel D

Private link:

<https://figshare.com/s/ede29446d6ad2d124727>

Supplement: Supplementary file 9 — Source Data for Figure 4 [file EMMM-10-e8446-s007.pdf]
