# Supplementary material for: Co‐targeting BET and MEK as salvage therapy for MAPK and checkpoint inhibitor‐resistant melanoma
Source: EMBO Mol Med. 2018 Apr 11;10(5):e8446. doi: 10.15252/emmm.201708446 (PMC5938620; doi:10.15252/emmm.201708446)
Supplement: Supplementary file 6 — Source Data for Figure 1 [file EMMM-10-e8446-s004.pdf]

Figure 1, panel E
Flow cytometry data corresponding to Figure 1, panel E

Link:
https://figshare.com/s/07bc43897b9ce2b191bb
