# Supplementary material for: Co‐targeting BET and MEK as salvage therapy for MAPK and checkpoint inhibitor‐resistant melanoma
Source: EMBO Mol Med. 2018 Apr 11;10(5):e8446. doi: 10.15252/emmm.201708446 (PMC5938620; doi:10.15252/emmm.201708446)
Supplement: Supplementary file 8 — Source Data for Figure 3 [file EMMM-10-e8446-s006.pdf]

Figshare.com

Figure 3, panel A

Private link:

For WM3000 spheroids
https://figshare.com/s/e0122d9b703e17b0869f

For M93-047 spheroids
https://figshare.com/s/dc0cc7542f6b1100c47e
