# Supplementary material for: Co‐targeting BET and MEK as salvage therapy for MAPK and checkpoint inhibitor‐resistant melanoma
Source: EMBO Mol Med. 2018 Apr 11;10(5):e8446. doi: 10.15252/emmm.201708446 (PMC5938620; doi:10.15252/emmm.201708446)
Supplement: Supplementary file 10 — Source Data for Figure 5 [file EMMM-10-e8446-s008.pdf]

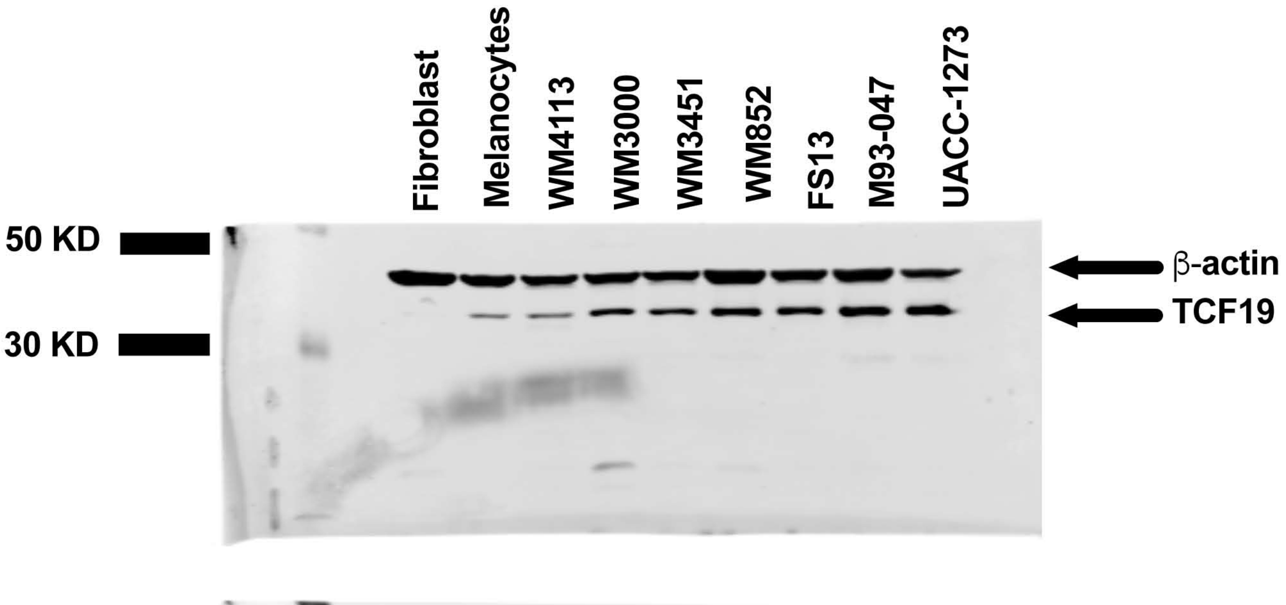
Fibroblast
Melanocytes
WM4113
WM3000
WM3451
WM852
FS13
M93-047
UACC-1273
50 KD
30 KD
β-actin
TCF19

Figshare.com

Figure 5, panel D

Private link:
https://figshare.com/s/2c18fbdd319a7fc3fefa

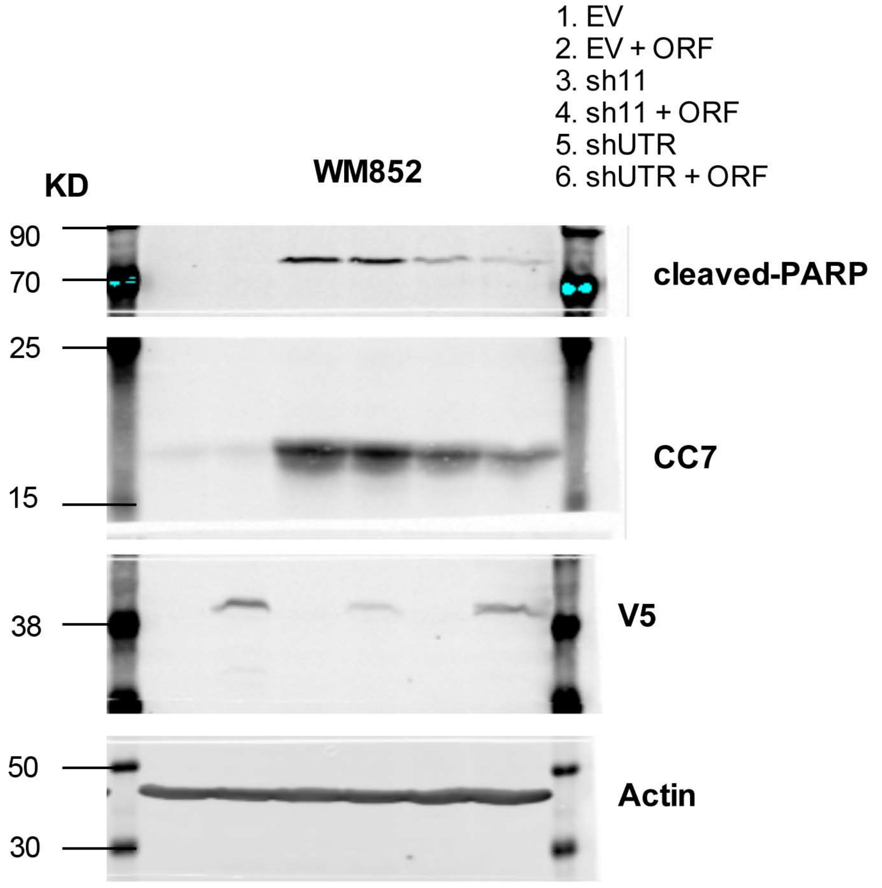
1. EV
2. EV + ORF
3. sh11
4. sh11 + ORF
5. shUTR
6. shUTR + ORF
WM852
KD
90
70
cleaved-PARP
25
15
CC7
38
V5
50
30
Actin
