# Supplementary material for: Co‐targeting BET and MEK as salvage therapy for MAPK and checkpoint inhibitor‐resistant melanoma
Source: EMBO Mol Med. 2018 Apr 11;10(5):e8446. doi: 10.15252/emmm.201708446 (PMC5938620; doi:10.15252/emmm.201708446)
Supplement: Supplementary file 11 — Source Data for Figure 6 [file EMMM-10-e8446-s009.pdf]

Figshare.com

Figure 6, panel H

Private link:
https://figshare.com/s/2dc565ba0417a8b4d92f
